# Primer and platform effects on 16S rRNA tag sequencing

**DOI:** 10.3389/fmicb.2015.00771

**Published:** 2015-08-04

**Authors:** Julien Tremblay, Kanwar Singh, Alison Fern, Edward S. Kirton, Shaomei He, Tanja Woyke, Janey Lee, Feng Chen, Jeffery L. Dangl, Susannah G. Tringe

**Affiliations:** ^1^Department of Energy Joint Genome InstituteWalnut Creek, CA, USA; ^2^National Research Council CanadaMontreal, QC, Canada; ^3^Illumina, Inc.San Francisco, CA, USA; ^4^Department of Biology and Howard Hughes Medical Institute, Curriculum in Genetics and Molecular Biology, Department of Microbiology and Immunology, Carolina Center for Genome Sciences, University of North CarolinaChapel Hill, NC, USA

**Keywords:** 16S rRNA gene sequencing, microbial population and community ecology, high throughput sequencing, microbial diversity, community assembly, amplification, sequencing error

## Abstract

Sequencing of 16S rRNA gene tags is a popular method for profiling and comparing microbial communities. The protocols and methods used, however, vary considerably with regard to amplification primers, sequencing primers, sequencing technologies; as well as quality filtering and clustering. How results are affected by these choices, and whether data produced with different protocols can be meaningfully compared, is often unknown. Here we compare results obtained using three different amplification primer sets (targeting V4, V6–V8, and V7–V8) and two sequencing technologies (454 pyrosequencing and Illumina MiSeq) using DNA from a mock community containing a known number of species as well as complex environmental samples whose PCR-independent profiles were estimated using shotgun sequencing. We find that paired-end MiSeq reads produce higher quality data and enabled the use of more aggressive quality control parameters over 454, resulting in a higher retention rate of high quality reads for downstream data analysis. While primer choice considerably influences quantitative abundance estimations, sequencing platform has relatively minor effects when matched primers are used. Beta diversity metrics are surprisingly robust to both primer and sequencing platform biases.

## Introduction

**Figure 1:**
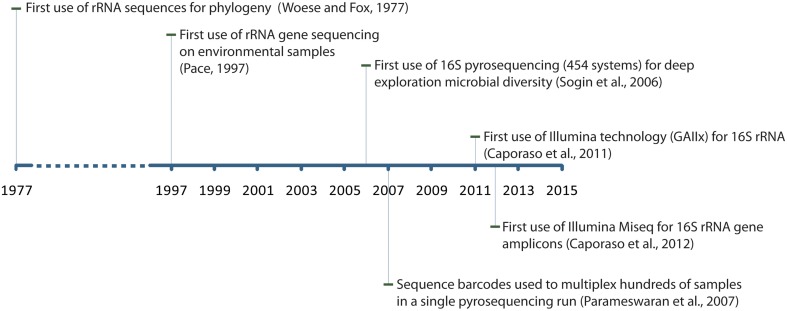
**Timeline indicating major breakthroughs in experimental and theoretical work in the field of 16S rRNA gene sequencing**.

Major breakthroughs in nucleic acids sequencing technology and molecular techniques over the last decades propelled the field of 16S rRNA gene sequencing as the backbone of modern microbial ecology (Figure 1). Carl Woese was the first to report using 16S rRNA genes as a marker for investigating bacterial phylogeny (Woese and Fox, 1977). His work provided a foundation for what would then become a new paradigm for microbial ecology. The following decades saw an extensive usage of the Sanger technology for sequencing of 16S rRNA genes which culminated with the demonstration that microorganisms could be studied (sequenced) directly in their environment without the need for cultivation in laboratory (Pace, 1997). This imprinted a lasting effect on our understanding of microbial diversity. With magnitude orders higher sequencing throughput, the 454 sequencing technology would eventually supersede Sanger systems for microbial population surveys by sequencing short 16S rRNA gene fragments (instead of full length rRNA genes) (Sogin et al., 2006) and allowed for multiplexing of hundreds of samples on a single sequencing run (Sogin et al., 2006; Parameswaran et al., 2007). The Illumina company later released an even higher throughput sequencing instrument (Genome Analyzer IIx) that largely outpaced 454 systems in term of throughput and reads quality and allowed the sequencing of highly multiplexed libraries (>100 samples) at a time (Caporaso et al., 2011). Today, the Illumina MiSeq system is solidly established as an instrument of choice for sequencing of 16S rRNA gene amplicons (Caporaso et al., 2012).

DNA sequencing of 16S rRNA genes or gene fragments has proven an effective method to inventory the microbial populations in a sample without the bias or effort of cultivation, and thus plays a key role in large ongoing microbial community studies such as the NIH funded human microbiome project (Human Microbiome Project Consortium, 2012a,b), the earth microbiome project (Gilbert et al., 2010) and plant microbiome studies (Mendes et al., 2011; Bulgarelli et al., 2012; Lundberg et al., 2012; Peiffer et al., 2013). Numerous microbial community surveys have relied on 454 pyrosequencing technology (pyrotags), due to its orders of magnitude higher throughput compared to its Sanger predecessor (Sogin et al., 2006; Tringe and Hugenholtz, 2008). Typically this involves amplifying short hypervariable regions from the 16S rRNA gene and including unique barcode tags in the primers, enabling highly multiplexed sequencing runs. In the past several years, the Illumina HiSeq and MiSeq sequencing platforms have surpassed 454 in terms of read quantity and quality and have been demonstrated to produce useful high-throughput 16S amplicon data as well (Caporaso et al., 2012), leading to their rapid adoption for tag sequencing.

In 16S tag sequencing experiments, it is accepted that a bias can be introduced by primer specificity as no primer pair is universal, and many studies have documented biases resulting from primer choice (Lee et al., 2012; Pinto and Raskin, 2012; He et al., 2013; Klindworth et al., 2013). Sequencing platform bias, on the other hand, is rarely considered, despite data demonstrating that significant bias can result from sequence features such as G+C content (Benjamini and Speed, 2012; Chen et al., 2013; Salipante et al., 2014); instead, sequencing platforms are considered primarily on the basis of features such as read length, error rate, throughput and cost. Comparisons between the data generated by different platforms have focused primarily on sequence quality metrics, data processing methods and classification accuracy (Claesson et al., 2010; Caporaso et al., 2012; Loman et al., 2012; Kozich et al., 2013; Nelson et al., 2014). Overall, these studies suggest that 16S rRNA data generated from different sequencing technologies should be readily comparable, but few address detailed taxonomic breakdown of the analyzed data or use PCR-independent data to assess bias.

Here we used the 454 and MiSeq platforms to sequence 16S tags amplified with primer pairs specific to the V4, V7–V8, and V6–V8 hypervariable regions from both defined microbial and environmental DNA samples. In addition, high depth shotgun sequencing (HiSeq) from unamplified DNA was performed for a selection of our environmental samples. 16S rRNA sequences were *in silico* extracted from these shotgun libraries for the purpose of having controls unaffected by amplification bias. We explored the correlation of taxonomic and diversity metrics between all data types. Our results cast some light on the impact of primer choice, sequencing platform and quality filtering on microbial community diversity metrics.

## Materials and methods

### Samples

DNA from various microbial organisms was pooled together at different concentrations (detailed in **Table 2**) to form what we refer to as our synthetic community. The final pool contained 160 ng/μl in 62.50 μl for a total of 10 μg. The *Pseudoxanthomonas suwonensis* single organism sample was prepared to a final concentration of 131 ng/μl. Expected distribution in the final mix was calculated (Equation 1) by first dividing the estimated quantity (μg) by the genome size for each organism which gave a value proportional to the number of genome copies added. Each of these values was then divided by the sum of genome copies from all organisms present in the synthetic community pool to get the final normalized proportions. Distribution percentages were also further normalized by the rRNA gene copy number for each organism. In that case, normalized rRNA gene copies added were obtained by dividing the quantity (μg) by the genome size and multiplying by rRNA gene copy number; each of these values was then divided by the sum of μg ∙ rRNA gene copy number divided by genome size of all organisms present in the synthetic community pool to get the final normalized proportions. rRNA gene copy number for each organism was determined using rnammer 1.2 (Lagesen et al., 2007).

(1) $$\begin{array}{l} MED(i)=\;\frac{\left(\frac{Q\left(i\right)}{GS\left(i\right)}\right)}{\left(\sum_{j=1}^{n=9}\frac{Q\left(j\right)}{GS\left(j\right)}\right)}\\ MEND(i)=\;\frac{\left(\frac{Q\left(i\right)\cdot \;RR(i)}{GS\left(i\right)}\right)}{\left(\sum_{j=1}^{n=9}\frac{Q\left(j\right)\;\cdot \;RR(j)}{GS\left(j\right)}\right)} \end{array}$$

**Equation 1**. Microorganism Expected Distribution (MED) and Microorganism Expected Normalized Distribution (MEND) equations for a given microorganism. *n* refers to the number of different microorganisms in the synthetic community. *Q*(*i*) is the DNA quantity added for organism *i, GS(i)* is the genome size of organism *i* in base pairs, and *RR(i)* is the rRNA gene copy number of organism *i*.

Wetland sediment samples were collected from a restored freshwater wetland in the Sacramento/San Joaquin Delta (Miller et al., 2008) using a Hargis corer sampling tool. Cores were dissected into bulk sediment and live root fractions and stored at −80°C until DNA extraction with a MoBio PowerLyzer PowerSoil kit according to manufacturer's instructions.

### Primer design, 16S amplification and sequencing procedures

Primer design for universal amplification of the V4 region of 16S rDNA was based on a protocol published by Caporaso and co-workers (Caporaso et al., 2011). The forward primer (515F) remained unchanged and the reverse primer was largely similar to the Caporaso V4 indexed reverse primers (806R), but with 0–3 random bases and the Illumina sequencing primer binding site added between the amplification primer and the Illumina adapter sequence. We also used primer pairs targeting the V6–V8 and V7–V8 regions (926F-1392R and 1114F-1392R) (Engelbrektson et al., 2010; Lundberg et al., 2012). Our primer sequences and staggered sequencing strategy are described in detail in the supplementary methods (Additional File 2) and Figure S1 (Additional File 1).

For each sample (and each replicate for *P. suwonensis* and synthetic community), three separate 16S rRNA gene amplification reactions targeting a given hypervariable region were performed, pooled together, cleaned up using AMPureXP (Beckman Coulter) magnetic beads and quantified with the Qubit HS assay (Invitrogen). Some samples were also analyzed with a BioAnalyzer 2100 (Agilent) instrument to confirm appropriate amplicon size. Pooled amplicons were then diluted to 10 nM and quantified by qPCR. Illumina amplicon tag (i.e., Itag) sequencing was performed according to standard DOE Joint Genome Institute procedures. Briefly, a density of 500,000 clusters/mm^2^ was targeted on each MiSeq lane which was also spiked with ~25% of a PhiX control library. Four hundred and fifty four pyrotag sequencing was performed as described (Kunin et al., 2010). Basecalling was done using Illumina's Real Time Analysis (RTA) software version 1.14.21. Obtained BCL files were converted into QSeq format using Bcl2Qseq 1.9.3, then converted to fastqs.

### Processing, clustering and classification of sequenced reads

Sequences were analyzed through our JGI Itag analysis pipeline (Itagger) summarized in Figure S2 (Additional File 1). Based on quality score data (Additional File 1: Figure S3), reads were trimmed to a length of 220 bases for 454 reads, 150 bases for MiSeq V4 and V6–V8 and 150 or 170 bases for MiSeq V7–V8. Note that because of read quality issues, single instead of paired end reads were analyzed for V6–V8 MiSeq amplicons. V4 and V7–V8 MiSeq reads were assembled with the FLASH software (Magoc and Salzberg, 2011). Common sequence contaminants and PhiX spike-in reads were removed from raw sequences using a kmer matching tool (DUK; http://duk.sourceforge.net/). Using in-house Perl scripts, assembled amplicons were then trimmed to remove reverse primer sequences (staggered primer sequences appearing in reverse reads). We then filtered amplicon sequences with either lenient or stringent quality control (QC) parameters. For the lenient QC condition, only sequences having more than 5 Ns, average quality score lower than 30, or more than 10 nucleotides having a quality score lower than 15 were rejected. The stringent QC condition rejected sequences that had 1 N or more; had average quality scores lower than 33; or had more than 3 nucleotides with a quality score lower than 20. Unless stated otherwise, the stringent QC parameters were used.

OTU generation was done using a pipeline based on USEARCH's OTU clustering recommendations (http://www.drive5.com/usearch/manual/otu_clustering.html). Briefly, quality controlled sequences were dereplicated at 100% identity. These 100% identity clustered reads were then denoised at 99% identity using USEARCH (Edgar, 2010). Clusters of less than three reads were discarded and remaining clusters were scanned for chimeras using UCHIME, first in *de novo* mode then in reference mode (Edgar et al., 2011) using the Broad Institute's 16S rRNA gene Gold reference database (Institute^1^). Remaining clusters were clustered at 97% identity (USEARCH) to produce OTUs.

Taxonomy assignment of resulting OTUs was performed using the RDP classifier (Wang et al., 2007) with a modified Greengenes training set built from a concatenation of the Greengenes database (Desantis et al., 2006), Silva eukaryotes 18S r108 (Quast et al., 2013) and the full-length 16S rDNA sequence of each microorganism used in our synthetic community pool listed in **Table 2**. Hierarchical tree files were generated with in-house Perl scripts and used to generate training sets using the RDP classifier (v2.3) training set generator's functionality (Wang et al., 2007). With taxonomic lineages in hand, OTU tables were generated, filtered to exclude eukaryotes and rarefied to the least abundant sample (2893 reads) across all different conditions. These OTU tables were used for downstream analysis.

Diversity metrics were obtained by aligning OTU sequences on a Greengenes core reference alignment (Desantis et al., 2006) using the PyNAST aligner (Caporaso et al., 2010). Alignments were filtered to keep only the V4, V7–V8, or V6–V8 part of the alignment. A phylogenetic tree was built from the alignment with FastTree (Price et al., 2010). Alpha (observed species) and beta (weighted or unweighted UniFrac and Bray Curtis distances) diversity metrics and taxonomic classifications were computed using the QIIME software suite (Caporaso et al., 2010; Kuczynski et al., 2011). The Greengenes-Silva modified 16S database in fasta format, its corresponding RDP training set and the Greengenes core reference alignment used in this study are available on request. Final OTU tables are available in Additional File 4 in a compressed zip archive.

### Error rate estimation

To estimate 16S rRNA gene sequencing error generated by different sequencing technologies and different variable regions, a subsample of 10,000 raw reads for each *P. suwonensis* dataset from each library was individually aligned (MUSCLE v3.8.3.1) (Edgar, 2004a,b) against their 16S rRNA reference gene trimmed to include only the V4, V6–V8, or V7–V8 regions. These 10,000 raw reads were then passed through diverse quality control filters and individually aligned against their 16S rRNA reference gene as well. The aligned portion only of both query and subject reads was extracted from each alignment and evaluated for errors (insertions, deletions or substitutions). *P. suwonensis* contains more than one copy of the 16S rRNA gene with two slightly different sequences. Therefore, each read was aligned against both rRNA gene sequences and only the best alignment was kept.

### Metagenome shotgun sequencing and 16S read classification

For each sample, an Illumina library was constructed with a target insert size of 250 bp, and sequenced on the Illumina HiSeq 2000 platform to generate paired-end (2 × 150 bp) reads. One lane of HiSeq reads was generated for each sample, with total raw sequence ranging from 40 to 60 Gbp from each lane.

Community profiling of metagenomic libraries was performed by filtering each library for sequencing contaminants/adapters and identifying potential rRNA gene sequences using a kmer matching program (DUK; http://duk.sourceforge.net/). This step greatly reduced the number of reads to be analyzed in downstream steps. Potential rRNA gene reads were then merged with their mate pairs using FLASH (when possible). Reads that failed to assemble were trimmed using a sliding window approach: starting from the 5′ end, a window of 20 bases was progressively moved toward the 3′ region and reads were trimmed when the mean quality in that window was lower than Q30. Remaining single end and paired-end reads were then quality filtered using stringent quality filtering described above. Filtered reads of length higher or equal to 75 bases were classified individually with the RDP classifier using our rRNA gene training set. Final lineages were obtained by keeping the deepest lineage having a RDP value threshold of at least 0.50. Wetlands samples have been previously described in detail (He et al., 2015). WL01: bulk soil from sampling site A; WL02: Tule roots from sampling site A; WL07: Tule roots from sampling site B; WL11: Tule roots from sampling site L.

### Nucleotide sequence accession numbers

Raw sequence reads of the 16S rRNA gene amplicon data were submitted to the Sequence Read Archive (SRA) under accession no. SRP060004. Raw sequence reads for metagenomes are available under accession numbers SRX482087 (WL01), SRP010751 (WL02), SRP010730 (WL07), and SRX480816 (WL11).

## Results

### Sequence read quality in PCR amplicon libraries

High throughput sequencing of 16S rRNA gene amplicons is a process in which read quality generated by sequencing instruments is crucial. The 454 platform, though widely used to perform 16S rRNA gene microbial community surveys, is known to make errors when sequencing runs of two or more identical nucleotides, also known as homopolymers, due to the use of native rather than “protected” nucleotides for extension (Margulies et al., 2005; Huse et al., 2007). 16S rRNA gene amplicon sequencing using our 454 Titanium FLX instrument produced sequence quality scores generally comparable to what we observe on our Illumina MiSeq instrument up to position 200, but with a quality drop at position 100 (Additional File 1: Figure S3).

We encountered initial challenges in generating data of good quality using 16S rRNA gene amplicons as sequencing templates. One of these was the low sequence diversity in the first several bases sequenced, which is known to compromise base calling and sequence quality on the Illumina platform. A previously described MiSeq 16S protocol using a universal 16S V4 region primer pair employed a PhiX shotgun library as a “spike-in” to increase diversity and improve sequence quality (Caporaso et al., 2011). However, using this protocol, including 25% PhiX spike-in, produced reads 1 of good quality but reads 2 of very poor quality with an effective read length of about 60 bp (Additional File 1: Figure S4). Varying PhiX spike-in concentrations from 15 to 80% had little effect on read 2 quality. As an alternative method of increasing library diversity, we modified the V4 reverse primers by removing the linker and replacing it by 0, 1, 2, or 3 random nucleotides (Additional File 1: Figure S1) in each of the 96 indexed reverse primers (Additional File 2). This “staggered” strategy produced excellent read 2 quality (Additional File 1: Figure S4); others have reported improved quality with similar protocols (Faith et al., 2013; Kozich et al., 2013; Lundberg et al., 2013). Our final workflow for 16S rRNA gene tag sequencing included this staggered approach combined with a final PhiX spike-in concentration of ~25% and overall cluster density of 500 K/mm^2^. A similar protocol was also developed for amplification and sequencing of the V6–V8 and V7–V8 region of the 16S rRNA gene for direct comparison to 454 pyrotag data. For the 926F-1392R primer pair, however, read 1 was of consistently low quality regardless of PhiX spike-in %, cluster density or use of staggered forward primers (Additional File 1: Figure S3). In all cases, the quality scores of read 1 abruptly plunged to 0 in the transition from position 28 (absolute pos. 947) to 29 (absolute pos. 948). That region is composed of a long stretch of Gs and Cs including a homopolymer of 6 G nucleotides (GGCGGGGGGCCGCCC) which corresponds to position 21–35 (absolute pos. 948–962). The Illumina sequencers are known to produce lower quality in regions of extreme G+C content and may have difficulty with long stretches of Gs or Cs, suggesting that sequencing from the 926F end was simply intractable. This could possibly result from a so-called “hard stop” due to secondary structure, a major challenge in sequencing high GC% regions (Hurt et al., 2012). We therefore used read 2 only for our analyses which corresponds to the V8 region and matches the single-direction reads produced in 454 pyrotag sequencing.

The 1114F-1392R primer pair (V7–V8), also employing a staggered reverse primer, produced reads 1 and 2 of good quality which could be readily overlapped and merged (Figure S3). Quality scores are generally higher for complex wetland sample libraries than for the simple *P. suwonensis* and synthetic community libraries, highlighting the challenge of generating good quality reads with low diversity samples (Figure S4, Additional File 1).

### Read filtering and recovery

**Figure 2:**
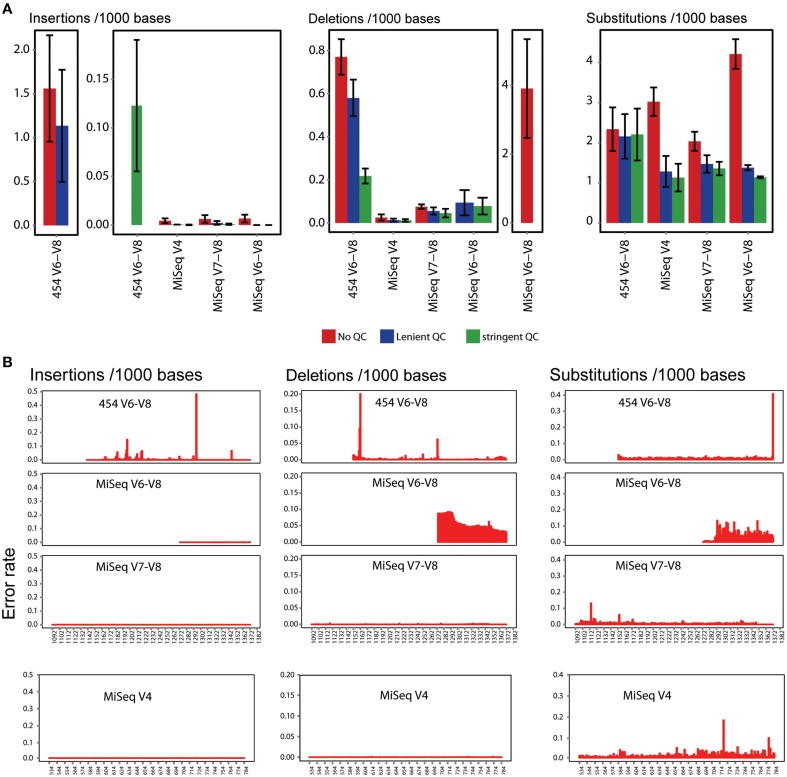
**Error estimation for various sequencing configurations. (A)** Insertion, deletion and substitution error frequency per 1000 *P. suwonensis* reads before and after lenient and stringent QC. Error frequency was calculated from triplicates for each sequencing condition. Error bars represent standard deviation. **(B)** Position of insertion, deletion and substitution error frequency in 16S tag amplicon sequences in which no QC filter has been applied.

Quality filtering is a critical step in 16S tag analysis, and for both 454 and early Illumina platforms others have found that aggressive quality control steps were necessary to reduce error, at times discarding more than half the raw data (Claesson et al., 2010; Caporaso et al., 2011; Degnan and Ochman, 2012). This is a concern, since sequence quality can depend on sequence composition and aggressive filtering could therefore bias results. To characterize sequencing errors introduced by both sequencing platforms and their reduction by QC filtering, we investigated the effects of filtering stringency on both error rates and calculated diversity metrics. We quantified the substitution, insertion and deletion error rates by aligning a random subset of 10,000 *P. suwonensis* reads generated by each sequencing platform and primer pair (V4, V7–V8, and V6–V8) on their 16S rRNA gene references and computed error types (see methods). Figure 2A shows that while introducing a QC step can significantly reduce error, the error rate reduction for MiSeq data is not significantly different between stringent and lenient QC, while for 454 V6–V8 reads insertions and deletions were highly reduced with stringent QC. Insertion error hotspots for 454 V6–V8 reads were observed at position 1298 and toward the end of the reads between positions 1221 and 1186 (Figure 2B). Many deletions occurred at positions 1165 and 1163 while substitutions were mainly observed at position 1372. Deletions and substitutions errors were observed at high frequencies throughout all the read length of V6–V8 sequences compared to the other data types.

As shown in Table 1, applying a stringent QC filter severely reduced the number of QC passed 454 reads which is not the case for MiSeq generated reads. In consequence, MiSeq data have far more usable reads (QC passed reads that can be used for downstream analyses). Read recovery was also generally higher for wetland sample libraries and lower for low complexity *P. suwonensis* and synthetic community samples. For all downstream analyses, we used stringent QC conditions.

**Table 1:** **Reads count summary of full datasets**.

**Library**	**Sample**	**Total reads (Read 1 + Read 2)**	**Assembled amplicons^b^**	**QC passed sequences**	**% passing QC^a^**
*P. suwonensis* MiSeq V4	*P. suwonensis* rep. #1	40,292+40,292	40,020	30,742	76.82%
	*P. suwonensis* rep. #2	27,867+27,867	27,656	20,775	75.12%
	*P. suwonensis* rep. #3	12,408+12,408	12,327	9,252	75.05%
Synthetic community MiSeq V4	Synthetic community rep. #1	37,758+37,758	37,496	26,608	70.96%
	Synthetic community rep. #2	47,646+47,646	47,303	34,115	72.12%
	Synthetic community rep. #3	59,307+59,307	58,906	41,560	70.55%
*P. suwonensis* MiSeq V6–V8	*P. suwonensis* rep. #1	0+126,349	–	101,907	80.66%
	*P. suwonensis* rep. #2	0+153,474	–	132,795	86.53%
	*P. suwonensis* rep. #3	0+180,811	–	157,568	87.15%
Synthetic community MiSeq V6–V8	Synthetic community rep. #1	0+135,496	–	113,949	84.10%
	Synthetic community rep. #2	0+158,396	–	134,772	85.09%
	Synthetic community rep. #3	0+203,480	–	164,724	80.95%
*P. suwonensis* MiSeq V7–V8	*P. suwonensis* rep. #1	275,924+275,924	275,156	180,808	65.71%
	*P. suwonensis* rep. #2	82,862+82,862	82,403	74,339	90.21%
	*P. suwonensis* rep. #3	391,600+391,600	390,326	355,689	91.13%
Synthetic community MiSeq V7–V8	Synthetic community rep. #1	74,930+74,930	74,197	67,501	90.98%
	Synthetic community rep. #2	359,731+359,731	358,811	326,660	91.04%
	Synthetic community rep. #3	397,267+397,267	395,589	354,364	89.58%
*P. suwonensis* 454 V6–V8	*P. suwonensis* rep. #1	42,694+0	–	16,003	37.48%
	*P. suwonensis* rep. #2	32,254+0	–	12,138	37.63%
	*P. suwonensis* rep. #3	22,015+0	–	8629	39.20%
Synthetic community 454 V6–V8	Synthetic community rep. #1	42,370+0	–	14,495	34.21%
	Synthetic community rep. #2	48,509+0	–	25,175	51.90%
	Synthetic community rep. #3	44,347+0	–	17,427	39.30%
Wetlands MiSeq V4	WL01	66,041+66,041	65,502	55,256	84.36%
	WL02	92,710+92,710	91,973	78,082	84.90%
	WL03	114,416+114,416	113,675	96,205	84.63%
	WL04	62,074+62,074	61,568	52,365	85.05%
	WL05	73,230+73,230	72,750	60,398	83.02%
	WL07	51,025+51,025	50,681	43,513	85.86%
	WL08	80,311+80,311	79,766	66,653	83.56%
	WL09	90,488+90,488	89,766	72,952	81.27%
	WL10	55,087+55,087	54,632	46,582	85.27%
	WL11	77,180+77,180	76,634	63,900	83.38%
Wetlands MiSeq V6–V8Wetlands MiSeq V7–V8	WL01	0+126,079	–	84,781	67.24%
	WL02	0+108,944	–	83,794	76.91%
	WL03	0+136,065	–	100,682	74.00%
	WL04	0+186,039	–	136,594	73.42%
	WL05	0+176,231	–	140,471	79.71%
	WL07	0+165,158	–	128,731	77.94%
	WL08	0+109,851	–	87,215	79.39%
	WL09	0+172,148	–	133,591	77.60%
	WL10	0+159,228	–	118,982	74.72%
	WL11	0+114,464	–	90,407	78.98%
	WL01	320,742+320,742	318,226	228,661	71.85%
	WL02	258,212+258,212	256,818	207,071	80.63%
	WL03	354,196+354,196	350,555	267,806	76.39%
	WL04	358,810+358,810	355,839	268,271	75.39%
	WL05	422,804+422,804	419,296	330,070	78.72%
	WL07	374,155+374,155	370,146	289,655	78.25%
	WL08	406,591+406,591	403,809	310,164	76.81%
	WL09	394,874+394,874	391,374	307,773	78.64%
	WL10	221,616+221,616	219,057	168,323	76.84%
	WL11	339,790+339,790	335,636	264,202	78.72%
Wetlands 454 V6–V8	WL02	25,977+0	–	12,823	49.36%
	WL03	18,852+0	–	9,202	48.81%
	WL04	50,363+0	–	22,863	45.40%
	WL05	17,490+0	–	8,482	48.50%
	WL07	15,617+0	–	7,431	47.58%
	WL08	16,173+0	–	7,462	46.14%
	WL09	13,899+0	–	6,597	47.46%
	WL10	33,079+0	–	15,746	47.60%
	WL11	9894+0	–	4849	49.01%

a *Reads were first filtered for Illumina adapter sequences and PhiX reads and separated by pairs. Disrupted pairs were discarded and remaining reads were binned by barcodes and processed through our stringent QC filter. QC passed reads were divided by these processed pre-QC reads to obtain percentage values*.

b *Pre-filtered assembled reads have slightly lower counts than their non-assembled counterparts because a small proportion of reads did not assemble*.

### Alpha diversity

**Figure 3:**
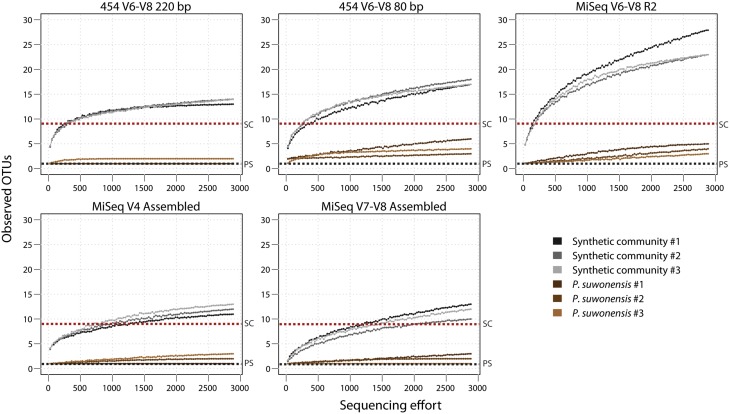
**Observed OTUs rarefaction estimation curves for *P. suwonensis* and synthetic community DNA pool**. A dotted black line shows the theoretical number of expected OTUs for *P. suwonensis* (PS) and a red line for the synthetic community (SC). All OTU tables used to generate rarefaction curves were rarefied to 2893 reads per sample.

Using our low complexity *P. suwonensis* and synthetic community libraries, we examined how alpha diversity behaved according to our different sequencing conditions. We first rarefied to the shallowest sample (which was 2893 read pairs) for all of our sequencing conditions and plotted an estimation of observed OTUs against sequencing effort for our various 16S tags (Rarefaction curves; Figure 3). For the V6–V8 tags, single-ended MiSeq reads retained just 80 bp after primer removal and showed higher observed OTUs than 220 and 80 bp equivalent tags sequenced with 454, suggesting the stringent QC was more effective in purging spurious OTUs from 454 data. We also plotted rarefaction curves for high complexity wetlands samples (Additional File 1: Figure S5 and found that V6–V8 amplicons showed reduced observed OTUs, regardless of sequencing technology, compared to V4 and V7–V8 tags. Since this was not observed in the synthetic community data, we hypothesize this could be due to greater conservation in this region of the 16S gene.

### Taxonomic classification relies more on primer specificity than on the sequencing platform

**Figure 4:**
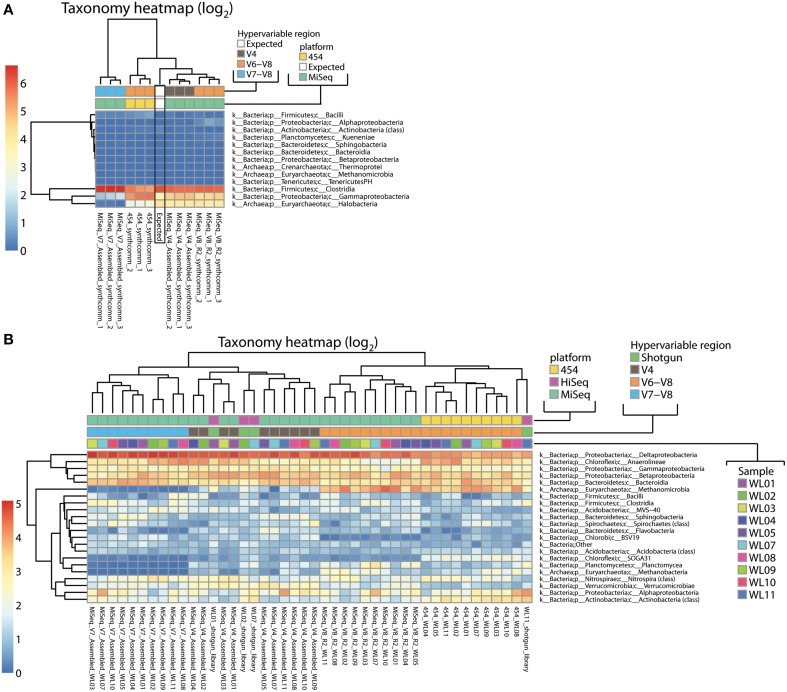
**Taxonomy heatmaps of all 16S data from (A) the synthetic community DNA pool and (B) samples from a wetlands sampling site**. Color scale is defined as log_2_ of percentage values of each taxon.

Using our rarefied OTU tables, we next evaluated the impact of primer choice and sequencing platform on phylogenetic classification as well as beta diversity metrics. Taxonomic distributions for the single species sample *P. suwonensis* are similar for all sequencing conditions and the vast majority of clusters point to the Gammaproteobacteria class. A few low abundance clusters were also found to point to Thermoprotei, Methanomicrobia and Clostridia (Additional File 1; Figure S6C). Figure 4A shows the classification obtained for our synthetic DNA pool of 9 different microorganisms described in Table 2. The classification profile shows a significant shift not only in classification patterns between the expected distribution and experimental classifications, but between different hypervariable region tags and sequencing platforms (Figure 4A). These biases were slightly more pronounced in the 454 V6–V8 tags compared to MiSeq V4 and V6–V8 tags. MiSeq V4 samples showed the highest similarity toward the expected taxonomic distribution. The largely bacteria-specific V7–V8 tags failed to amplify Halobacteria as expected, but also severely underrepresented Gammaproteobacteria and/or overrepresented Firmicutes. Alignments of the V4, V7–V8, and V6–V8 primer pairs against the corresponding region for the rRNA genes of each of the species present in the synthetic community show that none of the species contain mismatches to the degenerate V4 or V6–V8 primers, but there are lineage-specific variants that could affect melting temperature and therefore primer specificity (Additional File 1; Figure S6B). The V7 forward primer, by contrast, shows multiple mismatches to all three Euryarchaeota present in the synthetic community.

**Table 2:** **Synthetic community microorganism list and expected relative abundance**.

**Accession #**	**Microorganism name**	**Genome size (bp)**	**Quantity (μg)**	**rRNA gen copy**	**% of mix**	**Normalized % of mix by bp**	**Normalized % of mix by rRNA gene copy**	**Lineage**
NC_010001.1 3634491	*Clostridium phytofermentans* ISDg	4,847,594	3.50	8	35.00	30.37	71.35	Bacteria; Firmicutes; Clostridia; Clostridiales;Clostridium
CP003412.1 4091192	*Natrinema pellirubrum* str. J7-2	3,697,626	3.00	1	30.00	34.12	10.02	Archaea; Euryarchaeota; Halobacteria;Halobacteriales; Natrinema
AEDL00000000.1 4088228	*Pantoea* sp. AB-valens	4,368,708	1.50	1	15.00	14.44	4.24	Bacteria; Proteobacteria;Gammaproteobacteria; Enterobacteriales;Enterobacteriaceae; Pantoea
AGIL00000000.1 CP003470.1 4088401	*Rhodanobacter* sp. 2APBS1	4,225,490	1.00	2	10.00	9.95	5.85	Bacteria; Proteobacteria;Gammaproteobacteria; Xanthomonadales;Xanthomonadaceae; Rhodanobacter
AGIM00000000.1 CP003377.1 4091193	*Natronobacterium gregoryi* SP2	3,788,356	0.68	3	6.80	7.55	6.65	Archaea; Euryarchaeota; Halobacteria;Halobacteriales; Halobacteriaceae;Natronobacterium
CP002446.1 4090073	*Pseudoxanthomonas suwonensis* 11-1	3,419,049	0.20	2	2.00	2.46	1.45	Bacteria; Proteobacteria; Gammaproteobacteria; Xanthomonadales;Xanthomonadaceae; Pseudoxanthomonas
PRJNA60055 CP003078.1 4089496	*Mycobacterium smegmatis* JS623	6,464,916	0.06	2	0.60	0.39	0.23	Bacteria; Actinobacteria; Actinobacteria; Actinomycetales;Mycobacteriaceae; Mycobacterium
NZ_AGIR01000000 4090414 CP007060	*Halobacterium* sp. str DL1	2,846,968	0.04	1	0.40	0.59	0.17	Archaea; Euryarchaeota; Halobacteria;Halobacteriales; Halobacteriaceae;Halobacterium
AGIP00000000 4090068	*Paenibacillus lactis* 154	6,805,951	0.02	1	0.20	0.12	0.04	Bacteria; Firmicutes; Bacilli; Bacillales;Paenibacillaceae; Paenibacillus;Paenibacillus

Classification was also performed on environmental samples from a wetland sampling site (Miller et al., 2008) amplified with V4 (MiSeq), V7–V8 (MiSeq), and V6–V8 (both MiSeq and 454) primer pairs. Important variations in classification profiles were primarily observed between tags amplified with different primer pairs (V4, V7–V8, and V6–V8) rather than between the sequencing platforms (Figure 4B, Additional File 1; Figure S6A). Among MiSeq sequenced wetland tags, some clear discrepancies were apparent between primer sets. The most prominent was a near absence of Archaea in the V7–V8 datasets, an expected result of the mismatches to the forward primer, and a much higher abundance of Archaea in V6–V8 data than in V4 (Methanomicrobia and Methanobacteria). Most variations, such as higher representation of Sphingobacteria and Verrucomicrobiae in V4 datasets as compared to V7–V8 and V6–V8, are not clearly attributable to primer mismatches. Additionally, it is worth noting that samples from the same source DNA do not cluster together, demonstrating the large effects of primer and platform choice (Figure 4B).

When comparing V6–V8 data generated with different sequencing platforms, most differences involve poorly classified lineages such as “Other Bacteria,” Proteobacteria (higher abundance in MiSeq V6–V8) and Euryarchaeota, and thus are more readily explained by classification biases than by sequencing biases *per se*. Anaerolineae are consistently overrepresented in V6–V8 454 compared to MiSeq V6–V8 tags (Additional File 1: Figure S6A), possibly because tags from this underexplored family are assigned to low abundance taxonomic classes not considered in the shorter single-end V6–V8 MiSeq data.

To clarify the impact of primer bias and ascertain whether any set of primers is significantly more biased than others, we compared tag data with unamplified Illumina shotgun metagenome data, free of PCR bias, from a subset of the wetland samples. Potential rRNA gene reads were extracted from those libraries, paired-end assembled, trimmed, filtered and classified using the RDP classifier (Additional File 1: Table S2). Only reads classified as Bacteria or Archaea were used for comparison to tag libraries. Compared to the metagenome references, Methanomicrobia were underrepresented in MiSeq V4 and highly overrepresented in both 454 and MiSeq V6–V8 (and absent in V7–V8 which did not amplify archaea) (Additional File 1: Figure S6A). The opposite was observed for Deltaproteobacteria (overrepresentation in MiSeq V4 and V7–V8 and underrepresentation in 454 and MiSeq V6–V8). At the domain level, Archaea were heavily overrepresented in V6–V8 data regardless of platform. For instance, for the WL1 sample, relative abundance of archaeal organisms was 5.59% based on the metagenome reference, but 3–4-fold higher in V6–V8 data (20.11 and 16.74% on MiSeq and 454 respectively) and 4-fold lower in V4 data (1.34% on MiSeq). However, it is worth noting that the relative order of the samples in terms of archaeal abundance (WL7 < WL2 < WL1 < WL11) is preserved within each data type, suggesting that relative abundance between samples may still be qualitatively meaningful (Additional File 1: Figure S6A). When datasets were clustered based on class-level abundances, three of the shotgun datasets clustered with MiSeq V4 Illumina data and each other (Figure 4B). One outlier shotgun library was most similar to a 454 V6–V8 library from a different sample.

**Figure 5:**
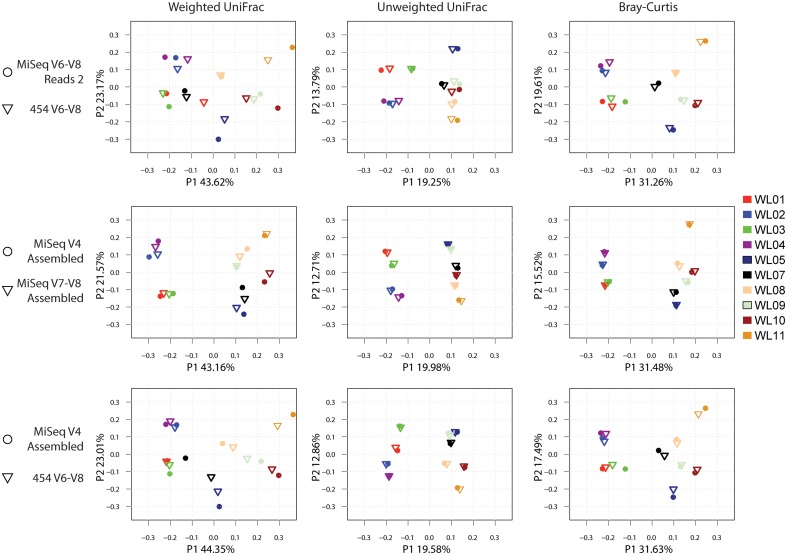
**Procrustes rotation comparison of weighted UniFrac, unweighted UniFrac and Bray-Curtis coordinates metrics for various wetland 16S tag data types**.

Previous studies have indicated that beta diversity metrics may be less sensitive to sequence error or primer bias than alpha diversity OTU richness metrics (Caporaso et al., 2012). Calculation of beta diversity metrics (Lozupone and Knight, 2005) followed by a Procrustes rotation (Gower, 1975) comparison of each wetland sample dataset supported this conclusion especially when using non-phylogenetic distance metrics (i.e., Bray-Curtis dissimilarity index) (Figure 5 and Table 3). Unweighted UniFrac and Bray-Curtis clustering patterns on PCoA plots were highly similar across all data types while weighted UniFrac exhibited greater variation. Accordingly, M^2^ rotation values are the highest for weighted UniFrac metrics, followed by Bray-Curtis and unweighted UniFrac (Figure 5).

**Table 3:** M^2^ and Monte Carlo *P*-values^*^ (10,000 permutations) Procrustes rotation comparison of weighted UniFrac, unweighted UniFrac and Bray-Curtis metrics.

	**MiSeq V4 assembled**	**MiSeq V7–V8 assembled**	**454 V6–V8**
**WEIGHTED UniFrac**			
MiSeq V7–V8 assembled	0.043 (0.0000)		
454 V6–V8	0.118 (0.0000)	0.123 (0.0000)	
MiSeq V6–V8 reads 2	0.209 (0.0004)	0.217 (0.0004)	0.200 (0.0004)
**UNWEIGHTED UniFrac**			
MiSeq V7–V8 assembled	0.004 (0.0000)		
454 V6–V8	0.008 (0.0000)	0.011 (0.0000)	
MiSeq V6–V8 reads 2	0.015 (0.0001)	0.010 (0.0000)	0.022 (0.0012)
**BRAY-CURTIS**			
MiSeq V7–V8 assembled	0.004 (0.0000)		
454 V6–V8	0.027 (0.0000)	0.025 (0.0000)	
MiSeq V6–V8 reads 2	0.031 (0.0000)	0.036 (0.0000)	0.038 (0.0000)

* *Monte Carlo P-values are in parentheses*.

## Discussion

“Staggered” primers with random bases inserted to increase complexity resulted in high quality 2X250 bp reads from V4 amplicons on MiSeq with minimal PhiX spike-in (Additional File 1: Figures S3, S4). Illumina has recently upgraded their Real Time Analysis software for basecalling (Illumina, 2014), improving performance for low diversity amplicon sequencing. This improvement allows the PhiX spike-in to be reduced to 5%, a standard sequencing control amount added to all libraries.

Assembled MiSeq amplicons have markedly lower insertion and deletion error rates than 454 reads (Figure 2A and Additional File 1: Table S1), resulting in higher read recovery rates after QC filtering. Even with high quality sequence data, bias due to primer specificity can interfere with accurate interpretation of 16S tag data (Lee et al., 2012; Pinto and Raskin, 2012; Klindworth et al., 2013). Polymerase error (Acinas et al., 2005), formation of chimeras (Qiu et al., 2001; Thompson et al., 2002; Kurata et al., 2004), multi-template amplification bias (Suzuki and Giovannoni, 1996; Polz and Cavanaugh, 1998) and primer mismatch (Baker et al., 2003; Huws et al., 2007; Sipos et al., 2007; Frank et al., 2008; Hong et al., 2009) can also compromise 16S-based studies and limit their utility.

In our study, PCR bias is directly demonstrated by the fact that the expected taxonomy and abundance of our simple synthetic DNA community does not exactly match what is experimentally observed with either V4 or V6–V8 16S tags. A heatmap of synthetic community distribution further exposes how well samples segregate according to their primer type (Figure 4A). Primer specificity bias is also exposed in natural environmental samples which show strong biases between V6 and V8 and all the other primer pairs (Figure 4B), none of which precisely matched profiles based on metagenome shotgun libraries made without PCR. Community biases related to hypervariable region choice within the 16S rRNA gene are increasingly being documented. One study investigated diversity of hypervariable regions *in silico* extracted from full length rRNA gene Sanger reads (Schloss, 2010). Hypervariable region alone (without the primer bias variable) was shown to introduce distortion into diversity metrics. A study comparing amplicons from V1 to V3, V4 to V6, and V7 to V9 hypervariable regions also showed differences in community compositions (Kumar et al., 2011). Another investigation of bias in amplicons generated with primers targeting V1–V3, V3–V5, and V6–V9 regions reported abundance discrepancies for certain phyla, which could at times be correlated with primer mismatches (Jumpstart Consortium Human Microbiome Project Data Generation Working Group, 2012). Another *in silico* approach attempted to benchmark various hypervariable regions in terms of diversity accuracy, suggesting that the V1–V3 and V4–V7 regions showed the shortest phylogenetic distance compared to full length rRNA sequences (Kim et al., 2011). Finally, amplifying similar rRNA hypervariable regions with two different primer pairs (V4–V6 vs. V6), then comparing only the shared V6 sequence, demonstrated variation in community composition based on protocol, but also high concordance between beta, and to a lesser extent alpha, diversity (He et al., 2013).

A sequencing platform (MiSeq vs. 454) bias is present as well with 454 data clustering together (Figure 3). Sequencing platform and primer biases were recently investigated in a study using primer pairs targeting V3–V4 and V4–V5 regions both on Illumina GAIIx and 454 FLX Titanium (Claesson et al., 2010). They found relative consistency between sequencing platforms, but found significant biases between both hypervariable region tags. Due to short reads length generated by the GAIIx platform, they also reported lower taxonomic classification resolution for this type of data.

It is worth noting that relative abundances among samples and beta diversity comparisons are often robust to all of these biases, as long as comparisons are confined to datasets generated with the same protocol (Figure 5 and Additional File 1: Figure S6A). Importantly, the fact that samples amplified from the same DNA source but with different primer pairs do not cluster together (Figure 4B; upper dendrogram) highlights the challenge in comparing amplicons obtained with different primer sets.

Sample contamination is something to consider as well: our MiSeq V6–V8 libraries were all sequenced in the same run, which likely explains unexpected classes in the Miseq V6–V8 synthetic community data (Figure 3A). Note the presence of Thermoprotei and Methanomicrobia taxa, which are found as contaminants in the *P. suwonensis* libraries as well, presumably due to cross-contamination by other samples or libraries.

There is currently no accepted consensus of what hypervariable region offers the “less” biased view of a bacterial community structure, as clearly no “perfect” hypervariable region exists. High-depth shotgun sequencing, while free of PCR primer bias, is still orders of magnitude more expensive than 16S amplicon sequencing for comparable 16S yields and is not yet a viable alternative to rRNA gene tags for community structure profiling. For the limited set of samples examined here, our data suggest that MiSeq V4 data are more similar to the shotgun libraries than other tag data generated. However, each sample will be different so it is important to be aware of the range and limitations of 16S rRNA gene primer pairs to appropriately amplify microorganisms of interest. It is therefore advisable to test primer pairs on samples of interest, and ideally compare to shotgun metagenome data, prior to performing large scale 16S tag sequencing surveys.

Procrustes rotation PCoA plots of various distance metrics (weighted and unweighted UniFrac and Bray-Curtis) (Figure 5) also highlights the challenges in comparing 16S tags from different hypervariable regions. Globally, clustering patterns were quite similar for all comparisons. However, a careful observation of spatial distribution shows that weighted UniFrac metrics, which take into account phylogenetic distances between samples and read abundance, have different clustering patterns between data from different experiments. In contrast, unweighted UniFrac and Bray-Curtis distances, which respectively consider phylogenetic distance and OTU abundance only, showed similar clustering patterns among various hypervariable regions and thus might be more appropriate metrics to compare different region tags.

Choosing appropriate QC parameters to minimize error while retaining sufficient data for statistical power is challenging, and the best choice will depend on sequencing technology and approach as well as run mode, run quality and intended analysis. Nevertheless, we found that whatever parameters we used for QC, MiSeq assembled reads consistently showed high recovery rates (and longest post-QC read length) due to the base correcting process occurring during overlapping paired-end assembly. Rarefaction curves of observed OTUs (Figure 3) show that our analysis pipeline managed to roughly capture the expected number of OTUs from our synthetic community and *P. suwonensis* samples, but alpha diversity is significantly affected by both sequence length and depth. Sequencing depth bias can be corrected using an appropriate cutoff for low abundance OTU filtering (i.e., filtering all OTUs having an abundance lower than 3 reads after the 99% identity clustering step greatly reduced spurious OTUs (Additional File 1: Tables S3–S5), but the threshold will differ for communities of varying complexity and sequencing of varying depth. In studies where the rare biosphere is of interest, such aggressive filtering may not be tolerable and sequence quality is therefore of even greater importance. While these concerns are hard to address by the study of synthetic communities, which lack the complexity of real environmental samples (Caporaso et al., 2011; Degnan and Ochman, 2012), the greater alpha diversity observed in natural samples with MiSeq assembled V4 data as compared to 454, even after stringent QC and especially when considering the greater read counts achievable with this technology, indicate this technology is preferred for rare biosphere applications.

## Conclusions

We have assessed the impact of primer choice and sequencing platform on 16S tag data from synthetic and natural microbial communities. Our data indicate that overlapping 250 bp paired-end MiSeq reads produce high-quality assembled amplicons amenable to stringent quality control parameters that lower spurious OTU cluster formation and thereby improve true novel OTU discovery. Primer choice has a much greater impact on biological results than sequencing platform, with V4 amplicons showing the greatest similarity to community profiles determined by shotgun sequencing. However, there are still important profile differences between amplicons and shotgun sequencing data and this in itself shows the limit of the 16S rRNA tag sequencing technology. While no primer set or sequencing platform produces quantitatively accurate population abundances or OTU counts, comparative analyses among samples with matched data types are largely robust to experimental methods used. Thus, protocol consistency, particularly with regard to primer choice, is more important in comparative 16S studies than the specific primers or platform used.

### Conflict of interest statement

The authors declare that the research was conducted in the absence of any commercial or financial relationships that could be construed as a potential conflict of interest.

## Footnotes

^1^Institute, B. *Microbiome Utilities* [Online]. Available: http://microbiomeutil.sourceforge.net/ [Accessed].
