# Supplementary material for: Primer and platform effects on 16S rRNA tag sequencing
Source: Front Microbiol. 2015 Aug 4;6:771. doi: 10.3389/fmicb.2015.00771 (PMC4523815; doi:10.3389/fmicb.2015.00771)
Supplement: Supplementary file 2 [file Additionalfile1.PDF]

a

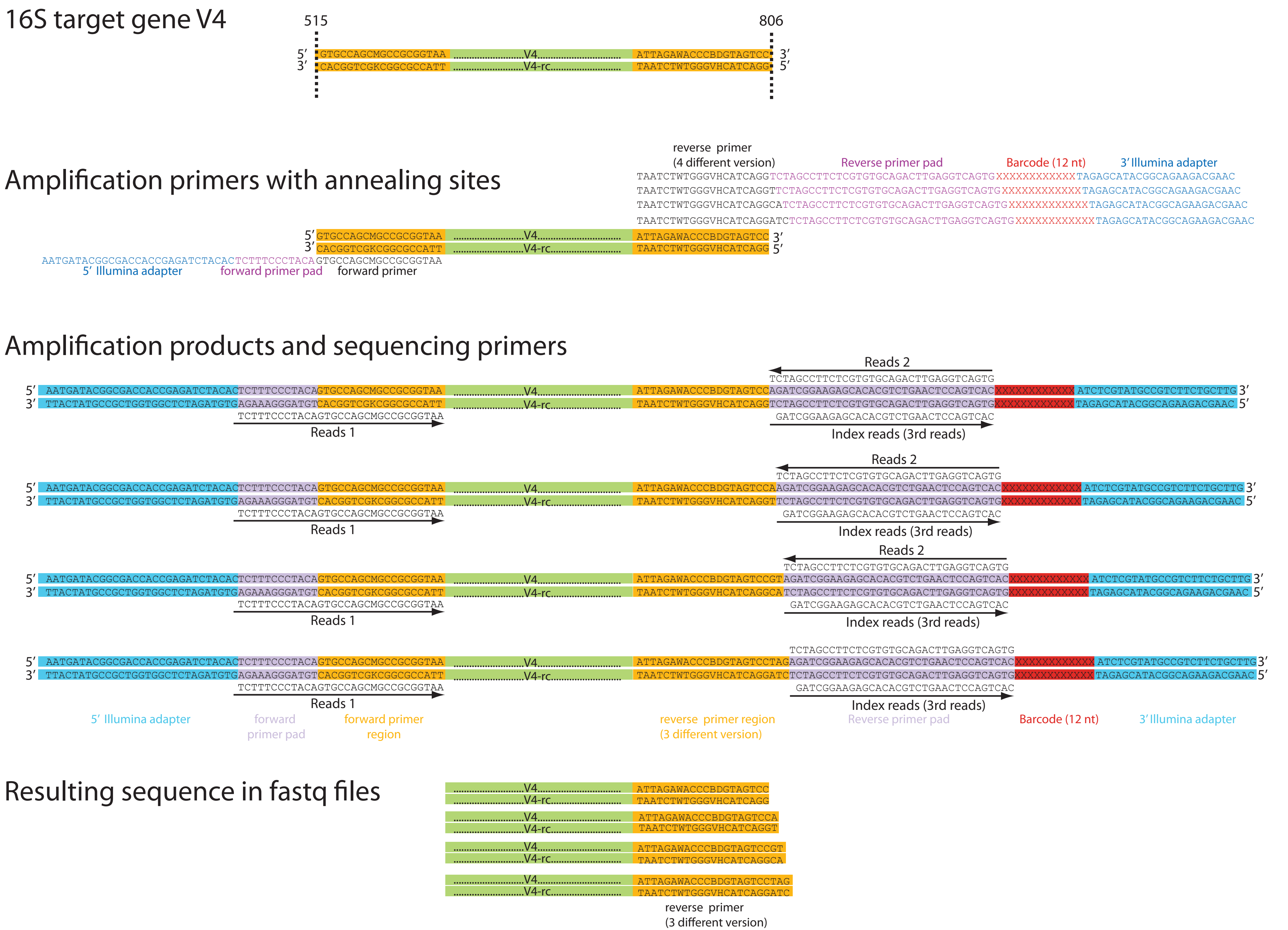

16S target gene V4
515
806
5′ GTGCCAGCMGCCGCGGTAA ...V4... ATTAGAWACCCBDGTAGTCC 3′
3′ CACGGTCGKCGGCGCCATT ...V4-rc... TAATCTWTGGGVHCATCAGG 5′
Amplification primers with annealing sites
reverse primer
(4 different version)
Reverse primer pad
Barcode (12 nt)
3′ Illumina adapter
TAATCTWTGGGVHCATCAGGTCTAGCCTTCTCGTGTGCAGACTTGAGGTCAGTGXXXXXXXXXXXXTAGAGCATACGGCAGAAGACGAAC
TAATCTWTGGGVHCATCAGGTTCTAGCCTTCTCGTGTGCAGACTTGAGGTCAGTGXXXXXXXXXXXXTAGAGCATACGGCAGAAGACGAAC
TAATCTWTGGGVHCATCAGGCATCTAGCCTTCTCGTGTGCAGACTTGAGGTCAGTGXXXXXXXXXXXXTAGAGCATACGGCAGAAGACGAAC
TAATCTWTGGGVHCATCAGGATCTCTAGCCTTCTCGTGTGCAGACTTGAGGTCAGTGXXXXXXXXXXXXTAGAGCATACGGCAGAAGACGAAC
5′ GTGCCAGCMGCCGCGGTAA ...V4... ATTAGAWACCCBDGTAGTCC 3′
3′ CACGGTCGKCGGCGCCATT ...V4-rc... TAATCTWTGGGVHCATCAGG 5′
AATGATACGGCGACCACCGAGATCTACACTCTTTCCCTACAGTGCCAGCMGCCGCGGTAA
5′ Illumina adapter
forward primer pad
forward primer
Amplification products and sequencing primers
Reads 2
TCTAGCCTTCTCGTGTGCAGACTTGAGGTCAGTG
5′ AATGATACGGCGACCACCGAGATCTACACTCTTTCCCTACAGTGCCAGCMGCCGCGGTAA ...V4... ATTAGAWACCCBDGTAGTCCAGATCGGAAGAGCACACGTCTGAACTCCAGTCACXXXXXXXXXXXXATCTCGTATGCCGTCTTCTGCTTG 3′
3′ TTACTATGCCGCTGGTGGCTCTAGATGTGAGAAAGGGATGTCACGGTCGKCGGCGCCATT ...V4-rc... TAATCTWTGGGVHCATCAGGTCTAGCCTTCTCGTGTGCAGACTTGAGGTCAGTGXXXXXXXXXXXXTAGAGCATACGGCAGAAGACGAAC 5′
TCTTTCCCTACAGTGCCAGCMGCCGCGGTAA
GATCGGAAGAGCACACGTCTGAACTCCAGTCAC
Reads 1
Index reads (3rd reads)
Reads 2
TCTAGCCTTCTCGTGTGCAGACTTGAGGTCAGTG
5′ AATGATACGGCGACCACCGAGATCTACACTCTTTCCCTACAGTGCCAGCMGCCGCGGTAA ...V4... ATTAGAWACCCBDGTAGTCCAAGATCGGAAGAGCACACGTCTGAACTCCAGTCACXXXXXXXXXXXXATCTCGTATGCCGTCTTCTGCTTG 3′
3′ TTACTATGCCGCTGGTGGCTCTAGATGTGAGAAAGGGATGTCACGGTCGKCGGCGCCATT ...V4-rc... TAATCTWTGGGVHCATCAGGTTCTAGCCTTCTCGTGTGCAGACTTGAGGTCAGTGXXXXXXXXXXXXTAGAGCATACGGCAGAAGACGAAC 5′
TCTTTCCCTACAGTGCCAGCMGCCGCGGTAA
GATCGGAAGAGCACACGTCTGAACTCCAGTCAC
Reads 1
Index reads (3rd reads)
Reads 2
TCTAGCCTTCTCGTGTGCAGACTTGAGGTCAGTG
5′ AATGATACGGCGACCACCGAGATCTACACTCTTTCCCTACAGTGCCAGCMGCCGCGGTAA ...V4... ATTAGAWACCCBDGTAGTCCGTAGATCGGAAGAGCACACGTCTGAACTCCAGTCACXXXXXXXXXXXXATCTCGTATGCCGTCTTCTGCTTG 3′
3′ TTACTATGCCGCTGGTGGCTCTAGATGTGAGAAAGGGATGTCACGGTCGKCGGCGCCATT ...V4-rc... TAATCTWTGGGVHCATCAGGCATCTAGCCTTCTCGTGTGCAGACTTGAGGTCAGTGXXXXXXXXXXXXTAGAGCATACGGCAGAAGACGAAC 5′
TCTTTCCCTACAGTGCCAGCMGCCGCGGTAA
GATCGGAAGAGCACACGTCTGAACTCCAGTCAC
Reads 1
Index reads (3rd reads)
TCTAGCCTTCTCGTGTGCAGACTTGAGGTCAGTG
5′ AATGATACGGCGACCACCGAGATCTACACTCTTTCCCTACAGTGCCAGCMGCCGCGGTAA ...V4... ATTAGAWACCCBDGTAGTCCTAGAGATCGGAAGAGCACACGTCTGAACTCCAGTCACXXXXXXXXXXXXATCTCGTATGCCGTCTTCTGCTTG 3′
3′ TTACTATGCCGCTGGTGGCTCTAGATGTGAGAAAGGGATGTCACGGTCGKCGGCGCCATT ...V4-rc... TAATCTWTGGGVHCATCAGGATCTCTAGCCTTCTCGTGTGCAGACTTGAGGTCAGTGXXXXXXXXXXXXTAGAGCATACGGCAGAAGACGAAC 5′
TCTTTCCCTACAGTGCCAGCMGCCGCGGTAA
GATCGGAAGAGCACACGTCTGAACTCCAGTCAC
Reads 1
Index reads (3rd reads)
5′ Illumina adapter
forward primer pad
forward primer region
reverse primer region (3 different version)
Reverse primer pad
Barcode (12 nt)
3′ Illumina adapter
Resulting sequence in fastq files
...V4... ATTAGAWACCCBDGTAGTCC
...V4-rc... TAATCTWTGGGVHCATCAGG
...V4... ATTAGAWACCCBDGTAGTCCA
...V4-rc... TAATCTWTGGGVHCATCAGGT
...V4... ATTAGAWACCCBDGTAGTCCGT
...V4-rc... TAATCTWTGGGVHCATCAGGCA
...V4... ATTAGAWACCCBDGTAGTCCTAG
...V4-rc... TAATCTWTGGGVHCATCAGGATC
reverse primer
(3 different version)

b

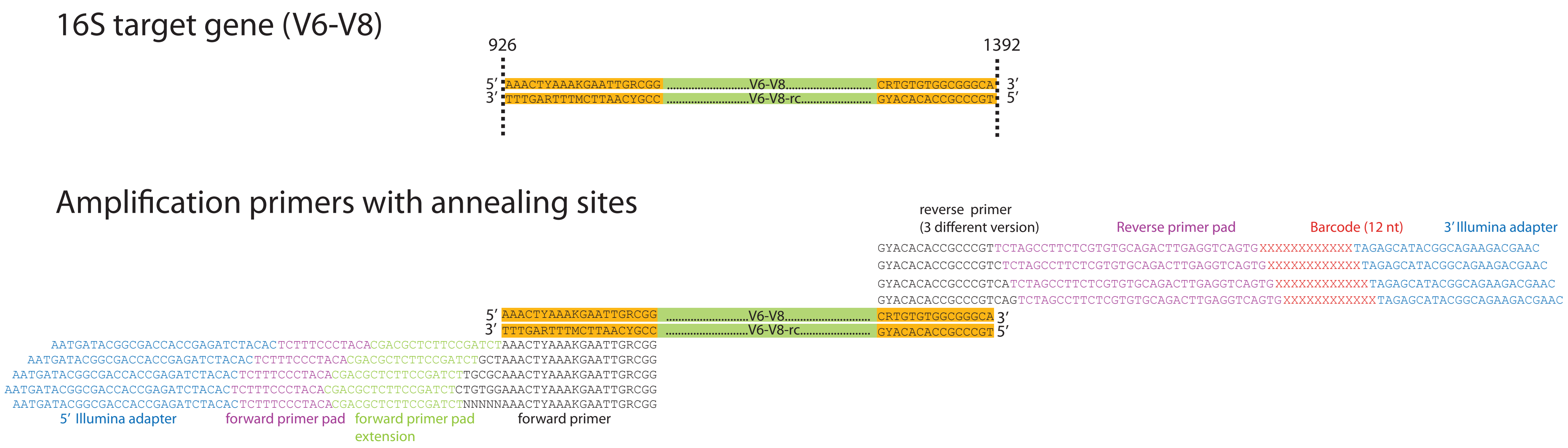

16S target gene (V6-V8)
926
1392
5′ AAACTYAAAKGAATTGRCGG ......V6-V8...... CRTGTGTGGCGGGCA 3′
3′ TTTGARTTTMCTTAACYGCC ......V6-V8-rc...... GYACACACCGCCCGT 5′
Amplification primers with annealing sites
reverse primer
(3 different version)
Reverse primer pad
Barcode (12 nt)
3′ Illumina adapter
GYACACACCGCCCGTTCTAGCCTTCTCGTGTGCAGACTTGAGGTCAGTGXXXXXXXXXXXXTAGAGCATACGGCAGAAGACGAAC
GYACACACCGCCCGTCTCTAGCCTTCTCGTGTGCAGACTTGAGGTCAGTGXXXXXXXXXXXXTAGAGCATACGGCAGAAGACGAAC
GYACACACCGCCCGTCATCTAGCCTTCTCGTGTGCAGACTTGAGGTCAGTGXXXXXXXXXXXXTAGAGCATACGGCAGAAGACGAAC
GYACACACCGCCCGTCAGTCTAGCCTTCTCGTGTGCAGACTTGAGGTCAGTGXXXXXXXXXXXXTAGAGCATACGGCAGAAGACGAAC
5′ AAACTYAAAKGAATTGRCGG ......V6-V8...... CRTGTGTGGCGGGCA 3′
3′ TTTGARTTTMCTTAACYGCC ......V6-V8-rc...... GYACACACCGCCCGT 5′
AATGATACGGCGACCACCGAGATCTACACTCTTTCCCTACACGACGCTCTTCCGATCTAAACTYAAAKGAATTGRCGG
AATGATACGGCGACCACCGAGATCTACACTCTTTCCCTACACGACGCTCTTCCGATCTGCTAAACTYAAAKGAATTGRCGG
AATGATACGGCGACCACCGAGATCTACACTCTTTCCCTACACGACGCTCTTCCGATCTTGCGCAAACTYAAAKGAATTGRCGG
AATGATACGGCGACCACCGAGATCTACACTCTTTCCCTACACGACGCTCTTCCGATCTCTGTGGAAACTYAAAKGAATTGRCGG
AATGATACGGCGACCACCGAGATCTACACTCTTTCCCTACACGACGCTCTTCCGATCTNNNNNAAACTYAAAKGAATTGRCGG
5′ Illumina adapter
forward primer pad
forward primer pad
extension
forward primer

c

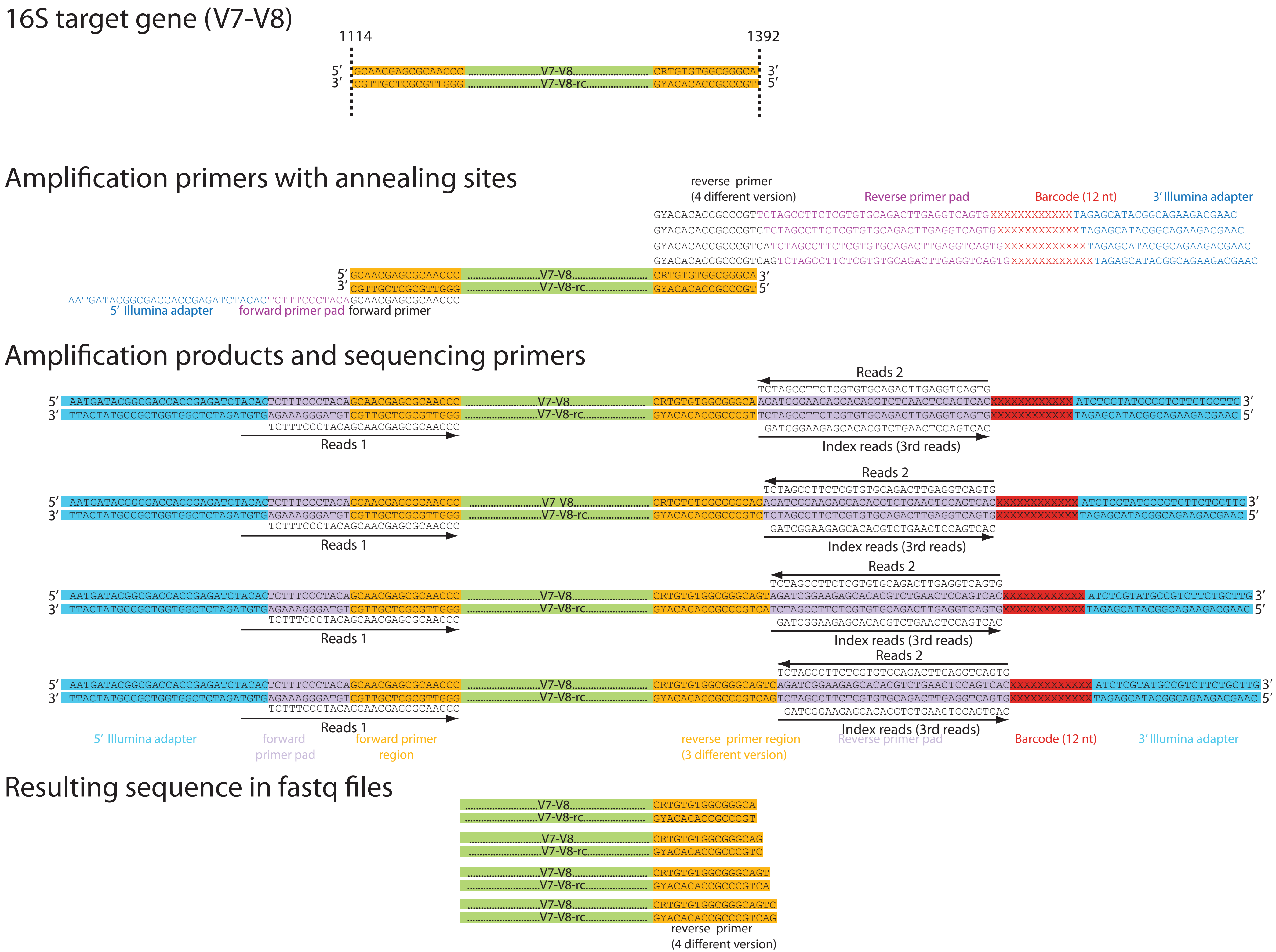

16S target gene (V7-V8)
1114
1392
5′ GCAACGAGCGCAACCC ....V7-V8.... CRTGTGTGGCGGGCA 3′
3′ CGTTGCTCGCGTTGGG ....V7-V8-rc.... GYACACACCGCCCGT 5′
Amplification primers with annealing sites
reverse primer
(4 different version)
Reverse primer pad
Barcode (12 nt)
3′ Illumina adapter
GYACACACCGCCCGTTCTAGCCTTCTCGTGTGCAGACTTGAGGTCAGTGXXXXXXXXXXXXTAGAGCATACGGCAGAAGACGAAC
GYACACACCGCCCGTCTCTAGCCTTCTCGTGTGCAGACTTGAGGTCAGTGXXXXXXXXXXXXTAGAGCATACGGCAGAAGACGAAC
GYACACACCGCCCGTCATCTAGCCTTCTCGTGTGCAGACTTGAGGTCAGTGXXXXXXXXXXXXTAGAGCATACGGCAGAAGACGAAC
GYACACACCGCCCGTCAGTCTAGCCTTCTCGTGTGCAGACTTGAGGTCAGTGXXXXXXXXXXXXTAGAGCATACGGCAGAAGACGAAC
5′ GCAACGAGCGCAACCC ....V7-V8.... CRTGTGTGGCGGGCA 3′
3′ CGTTGCTCGCGTTGGG ....V7-V8-rc.... GYACACACCGCCCGT 5′
AATGATACGGCGACCACCGAGATCTACACTCTTTCCCTACAGCAACGAGCGCAACCC
5′ Illumina adapter
forward primer pad
forward primer
Amplification products and sequencing primers
Reads 2
TCTAGCCTTCTCGTGTGCAGACTTGAGGTCAGTG
5′ AATGATACGGCGACCACCGAGATCTACACTCTTTCCCTACAGCAACGAGCGCAACCC ....V7-V8.... CRTGTGTGGCGGGCAAGATCGGAAGAGCACACGTCTGAACTCCAGTCACXXXXXXXXXXXX ATCTCGTATGCCGTCTTCTGCTTG 3′
3′ TTACTATGCCGCTGGTGGCTCTAGATGTGAGAAAGGGATGTCGTTGCTCGCGTTGGG ....V7-V8-rc.... GYACACACCGCCCGTTCTAGCCTTCTCGTGTGCAGACTTGAGGTCAGTGXXXXXXXXXXXXTAGAGCATACGGCAGAAGACGAAC 5′
TCTTTCCCTACAGCAACGAGCGCAACCC
GATCGGAAGAGCACACGTCTGAACTCCAGTCAC
Reads 1
Index reads (3rd reads)
Reads 2
TCTAGCCTTCTCGTGTGCAGACTTGAGGTCAGTG
5′ AATGATACGGCGACCACCGAGATCTACACTCTTTCCCTACAGCAACGAGCGCAACCC ....V7-V8.... CRTGTGTGGCGGGCAGAGATCGGAAGAGCACACGTCTGAACTCCAGTCACXXXXXXXXXXXX ATCTCGTATGCCGTCTTCTGCTTG 3′
3′ TTACTATGCCGCTGGTGGCTCTAGATGTGAGAAAGGGATGTCGTTGCTCGCGTTGGG ....V7-V8-rc.... GYACACACCGCCCGTCTCTAGCCTTCTCGTGTGCAGACTTGAGGTCAGTGXXXXXXXXXXXXTAGAGCATACGGCAGAAGACGAAC 5′
TCTTTCCCTACAGCAACGAGCGCAACCC
GATCGGAAGAGCACACGTCTGAACTCCAGTCAC
Reads 1
Index reads (3rd reads)
Reads 2
TCTAGCCTTCTCGTGTGCAGACTTGAGGTCAGTG
5′ AATGATACGGCGACCACCGAGATCTACACTCTTTCCCTACAGCAACGAGCGCAACCC ....V7-V8.... CRTGTGTGGCGGGCAGTAGATCGGAAGAGCACACGTCTGAACTCCAGTCACXXXXXXXXXXXX ATCTCGTATGCCGTCTTCTGCTTG 3′
3′ TTACTATGCCGCTGGTGGCTCTAGATGTGAGAAAGGGATGTCGTTGCTCGCGTTGGG ....V7-V8-rc.... GYACACACCGCCCGTCATCTAGCCTTCTCGTGTGCAGACTTGAGGTCAGTGXXXXXXXXXXXXTAGAGCATACGGCAGAAGACGAAC 5′
TCTTTCCCTACAGCAACGAGCGCAACCC
GATCGGAAGAGCACACGTCTGAACTCCAGTCAC
Reads 1
Index reads (3rd reads)
Reads 2
TCTAGCCTTCTCGTGTGCAGACTTGAGGTCAGTG
5′ AATGATACGGCGACCACCGAGATCTACACTCTTTCCCTACAGCAACGAGCGCAACCC ....V7-V8.... CRTGTGTGGCGGGCAGTCAGATCGGAAGAGCACACGTCTGAACTCCAGTCACXXXXXXXXXXXX ATCTCGTATGCCGTCTTCTGCTTG 3′
3′ TTACTATGCCGCTGGTGGCTCTAGATGTGAGAAAGGGATGTCGTTGCTCGCGTTGGG ....V7-V8-rc.... GYACACACCGCCCGTCAGTCTAGCCTTCTCGTGTGCAGACTTGAGGTCAGTGXXXXXXXXXXXXTAGAGCATACGGCAGAAGACGAAC 5′
TCTTTCCCTACAGCAACGAGCGCAACCC
GATCGGAAGAGCACACGTCTGAACTCCAGTCAC
Reads 1
Index reads (3rd reads)
5′ Illumina adapter
forward primer pad
forward primer region
reverse primer region (3 different version)
Reverse primer pad
Barcode (12 nt)
3′ Illumina adapter
Resulting sequence in fastq files
....V7-V8.... CRTGTGTGGCGGGCA
....V7-V8-rc.... GYACACACCGCCCGT
....V7-V8.... CRTGTGTGGCGGGCAG
....V7-V8-rc.... GYACACACCGCCCGTC
....V7-V8.... CRTGTGTGGCGGGCAGT
....V7-V8-rc.... GYACACACCGCCCGTCA
....V7-V8.... CRTGTGTGGCGGGCAGTC
....V7-V8-rc.... GYACACACCGCCCGTCAG
reverse primer
(4 different version)

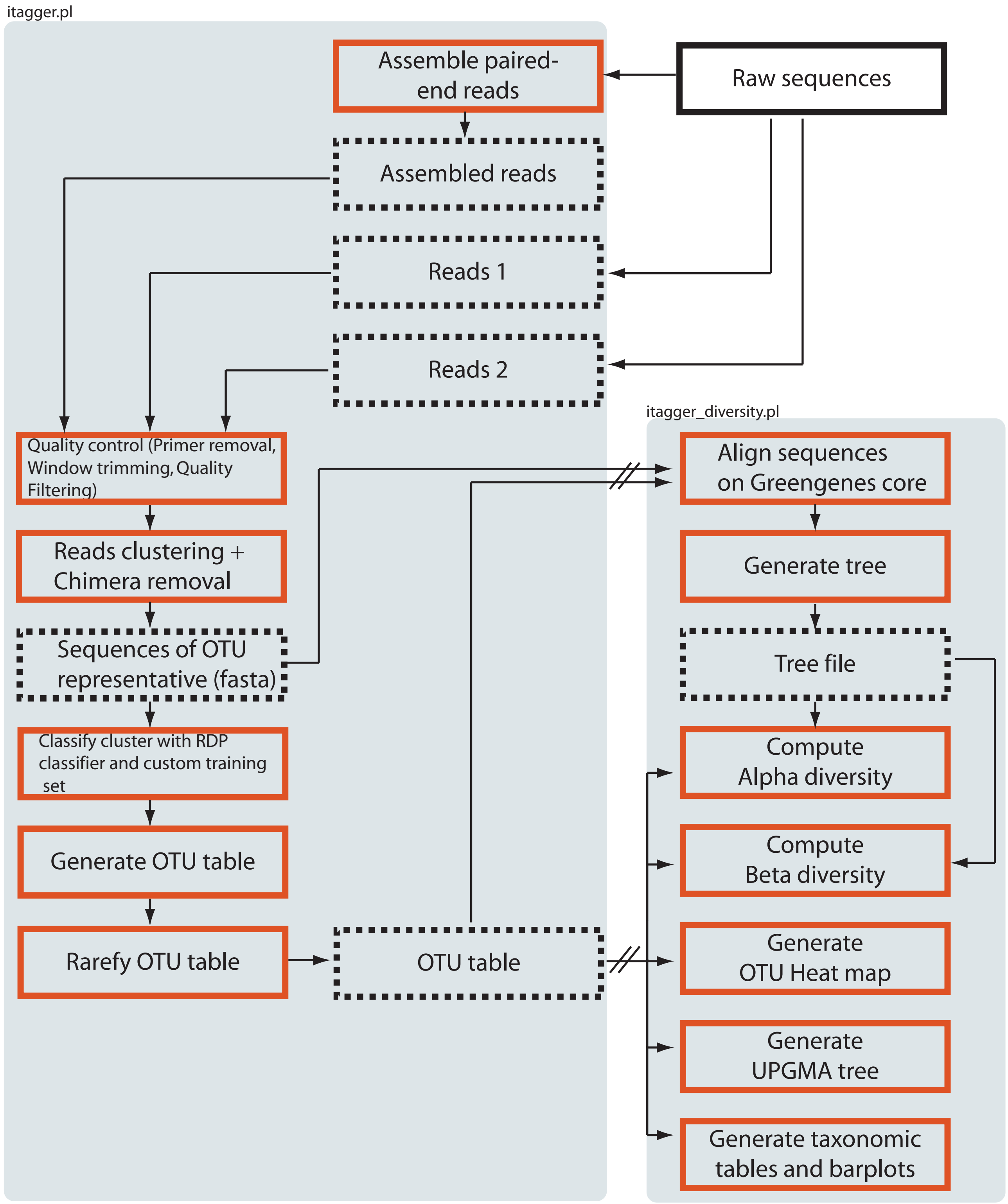

Supplemental figure S2. Itagger pipeline schematics. Dashed lined boxes represent important files generated throughout the pipeline. Analysis pipeline is divided in two main parts. First, raw sequences are processed and clustered to generate an OTU table (itagger.pl). Using multiple OTU tables as input, various diversity metrics are then computed.

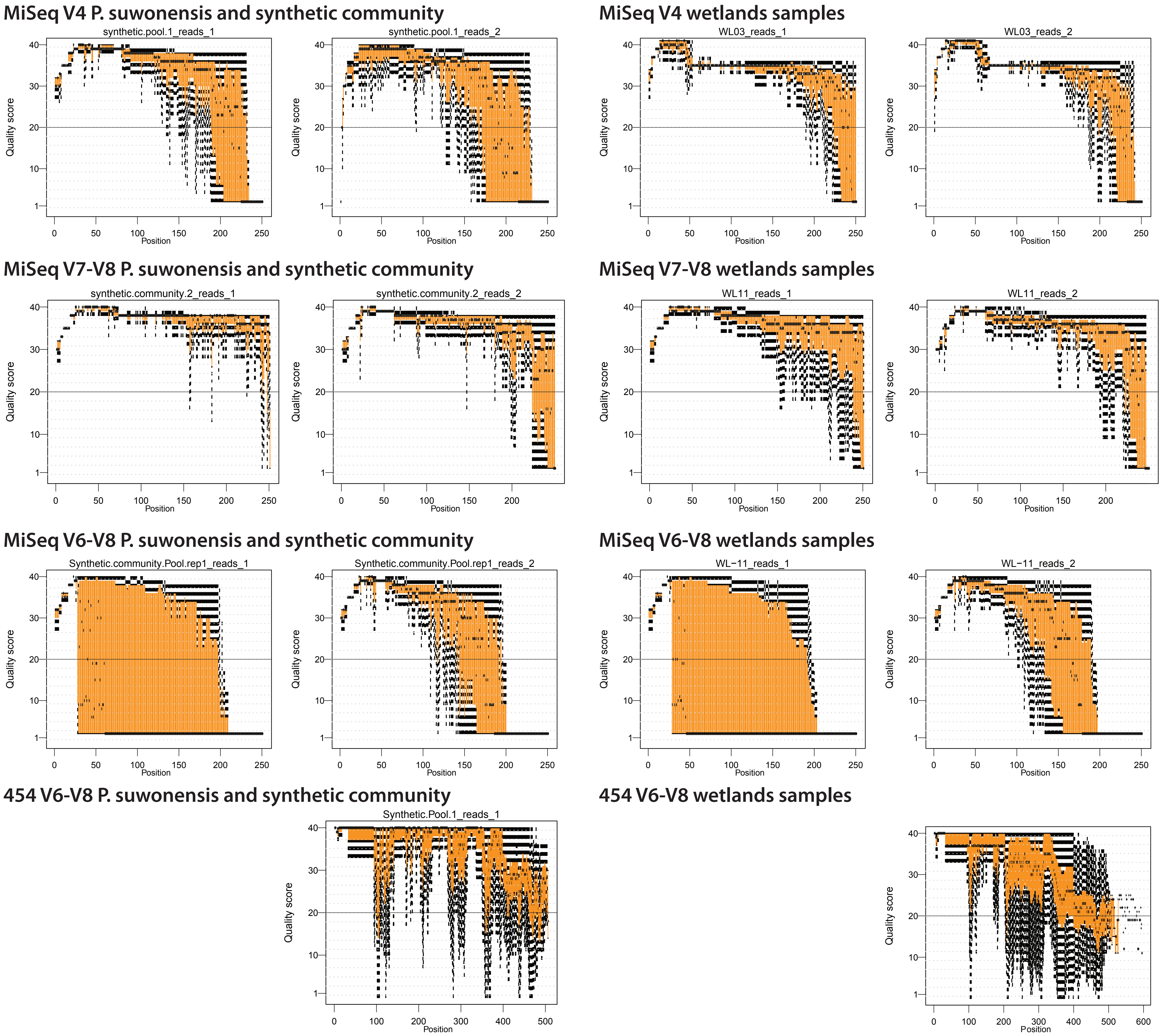

Figure S3. Quality score plots representative for each condition. A meaningful representative quality score plot was chosen among all samples/barcodes for each sequencing condition tested in this study. Orange bars represent the Interquartile range.

a Standard(non-staggered) V4 primers

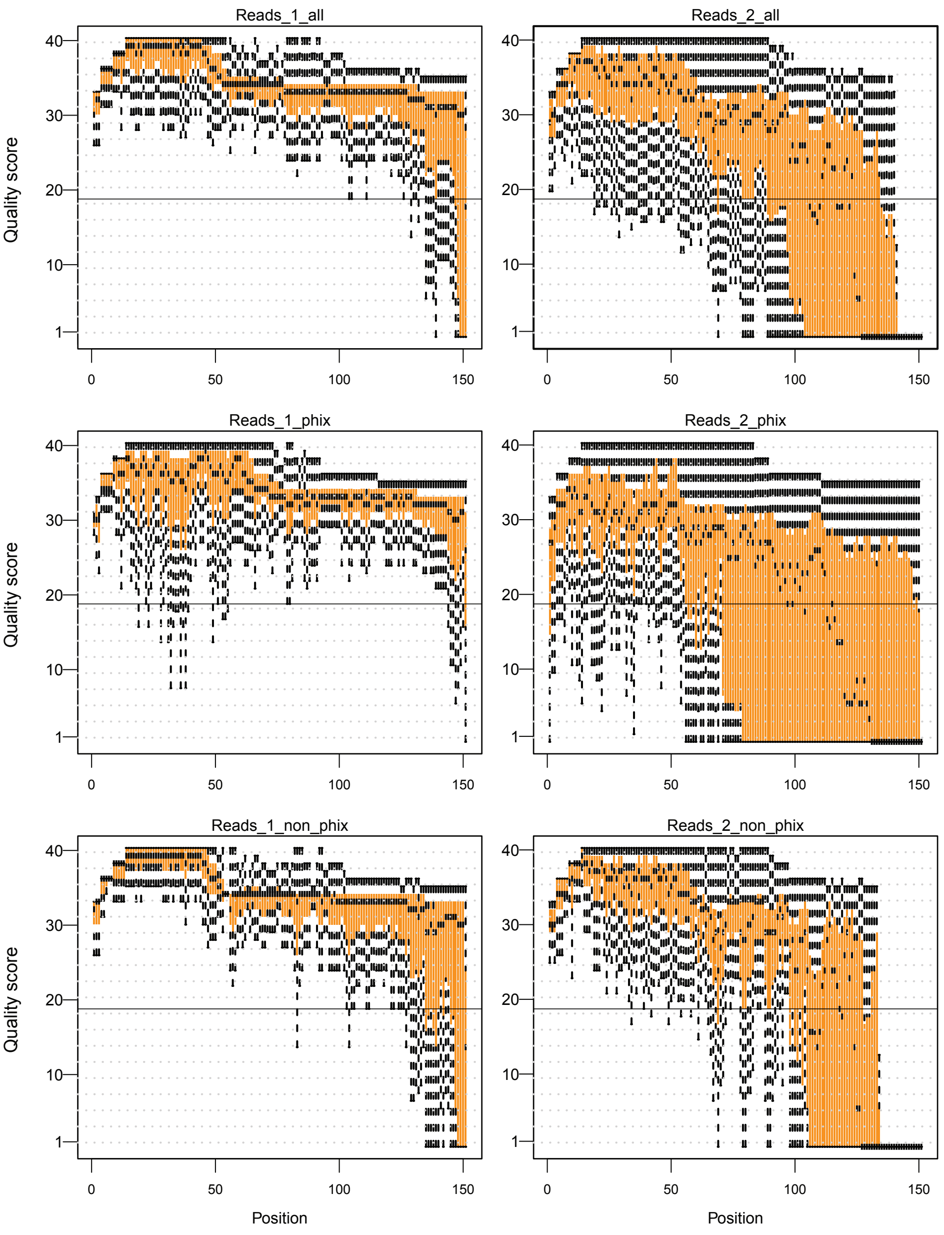

b Staggered V4 primers

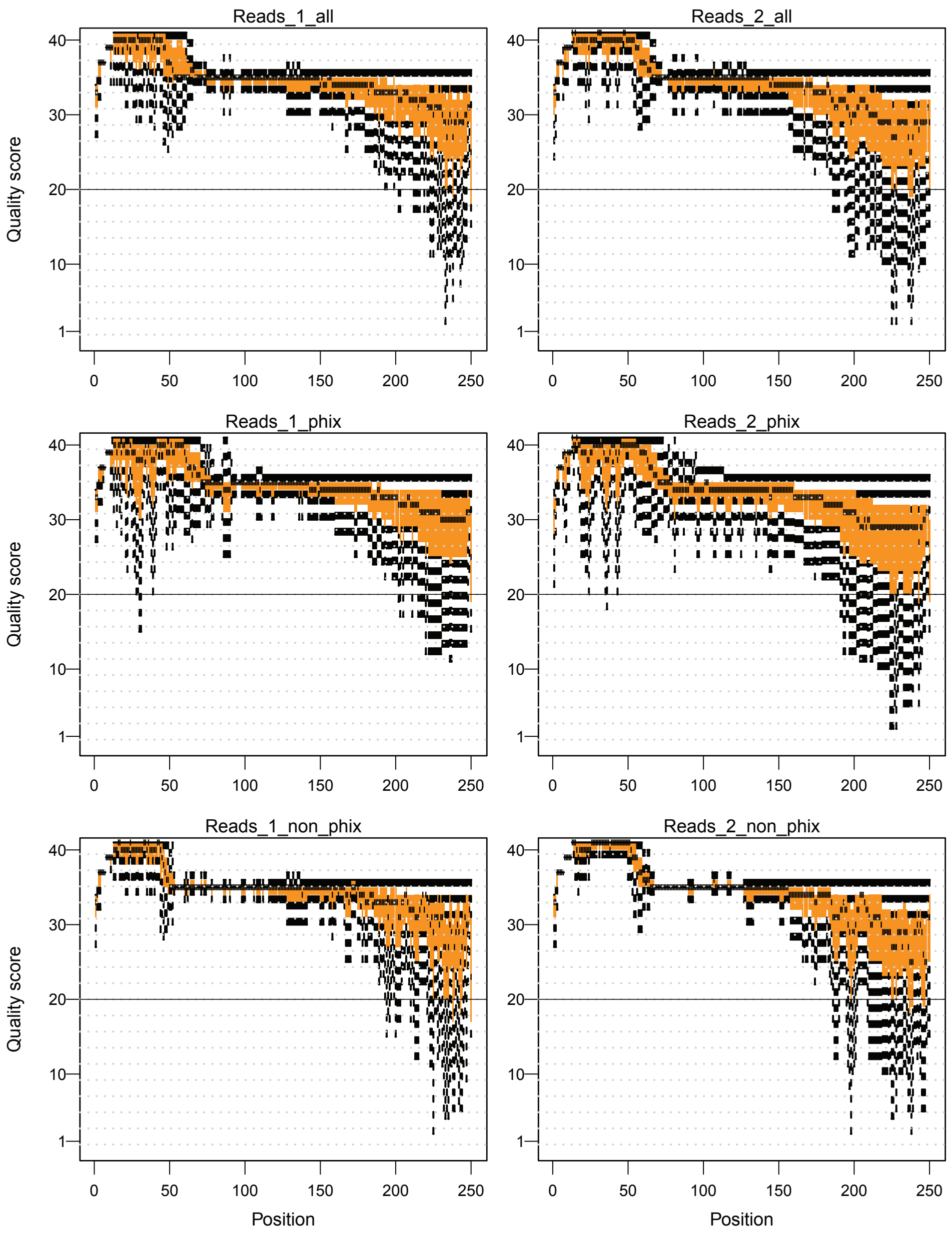

c Typical quality plot of pyrotags obtained with 454 Ti FLX sequencer

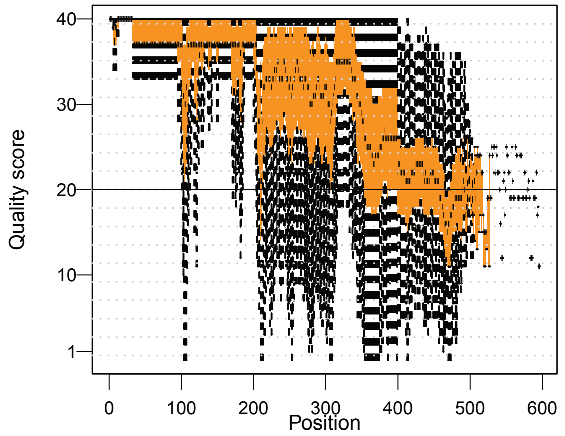

Figure S4. Boxplots of quality score plots for staggered vs non-staggered amplicons for V4 hypervariable region amplified from wetlands samples. Each sequencing run a) staggered and b) non-staggered amplicons) represent a complete MiSeq lane. Quality score plots were generated for all reads, reads 1 and reads 2 and were also split (and plotted) as PhiX and non-PhiX reads. Quality score plots of reads sequenced with 454 Ti FLX are also shown (c). Orange bars represent the Interquartile range.

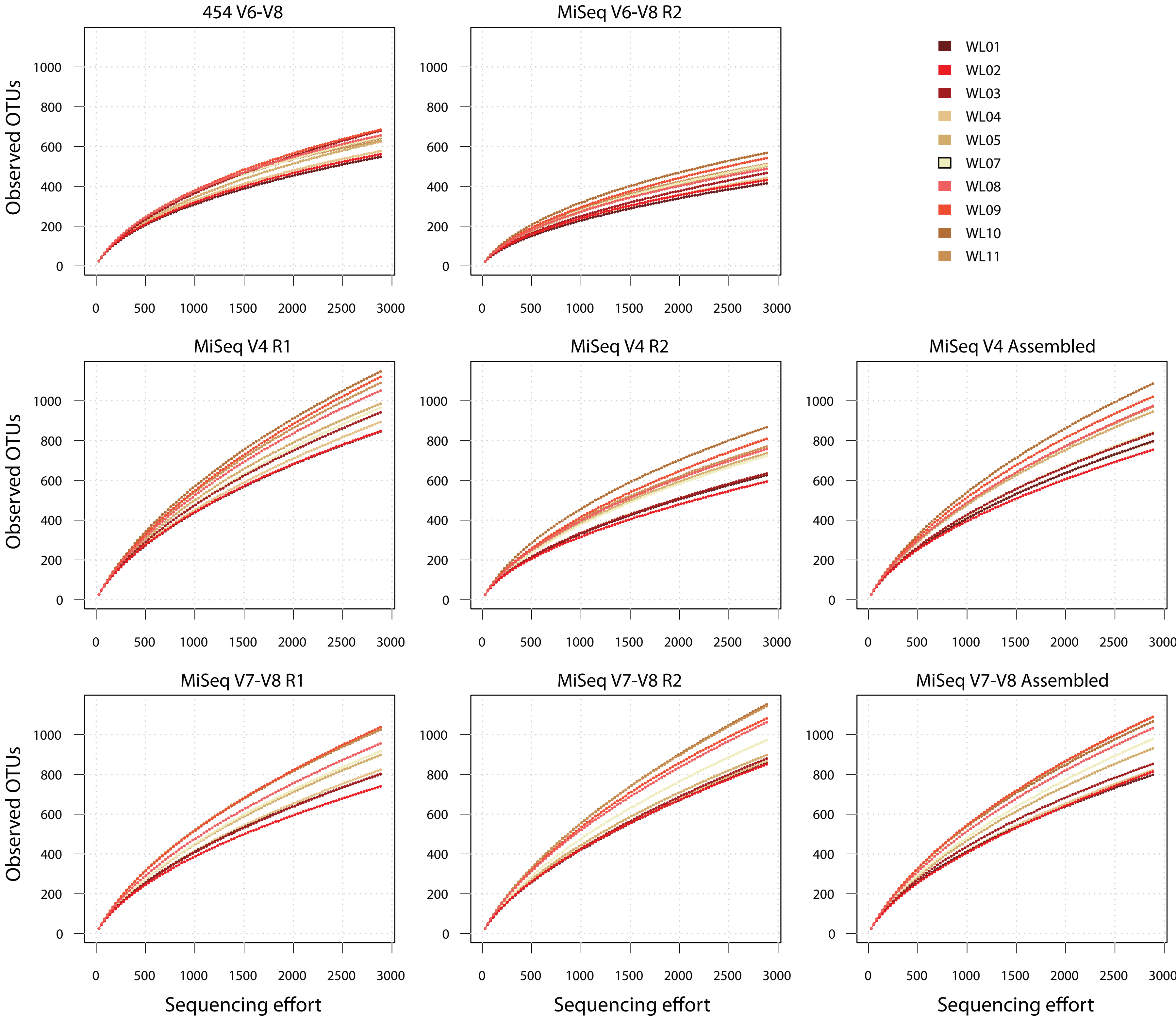

Figure S5. Alpha diversity rarefaction curves (Observed OTUs) for wetlands samples.

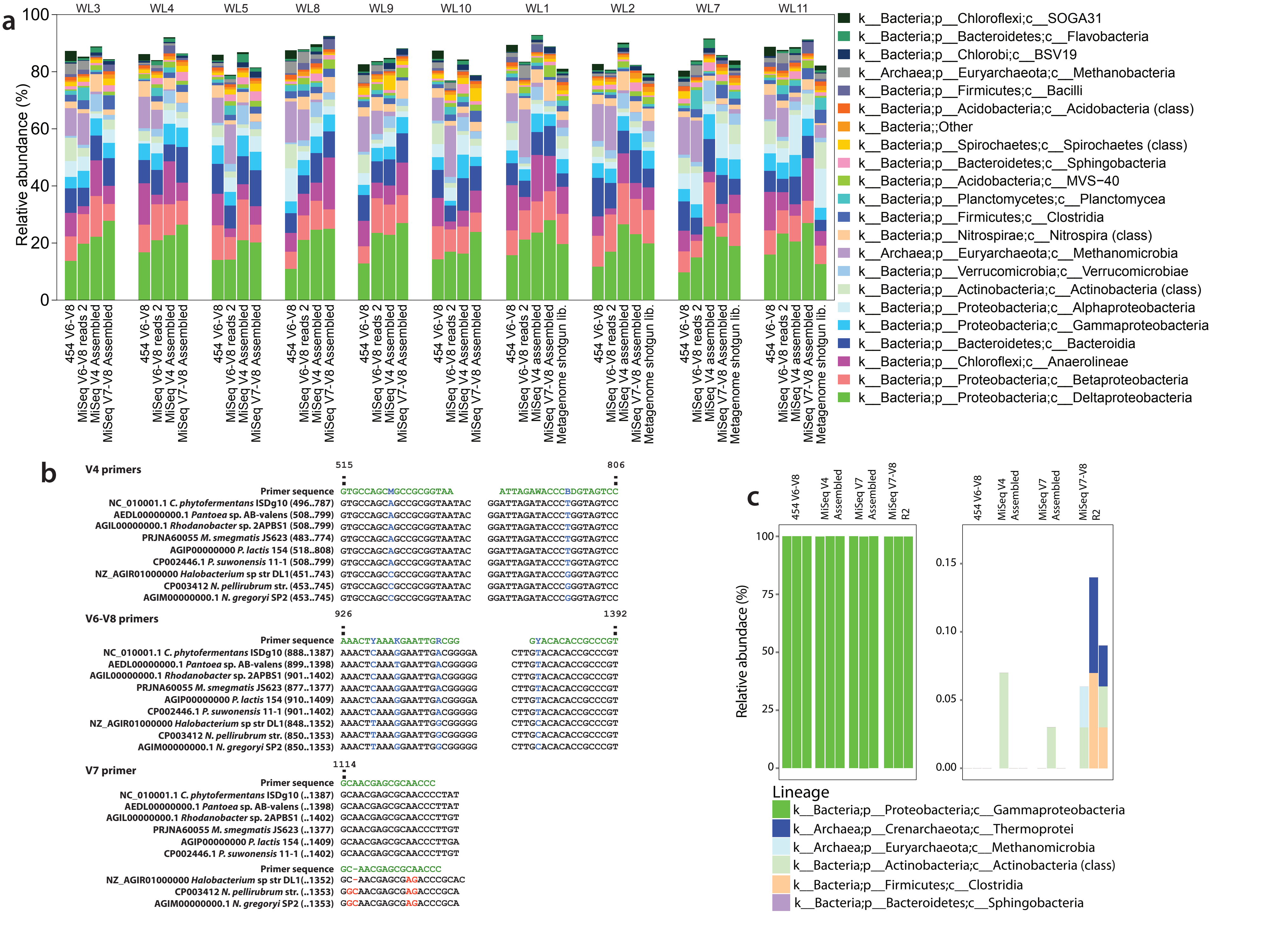

Figure S6. Taxonomic classification at the Class depth for **(a)** samples of a wetlands sampling site. Taxonomy was established for the 22 most abundant OTUs. **b)** Alignments of V4, V6-V7 and V6-V8 primer pairs against synthetic community 16S rRNA annealing regions. Red=mismatch, blue=ambiguous nucleotide match. **c)** Classification of OTUs at the Class depth for samples containing *P. suwonensis* species. For ambiguous nucleotides; M=A or C; B=C or G or T; R=A or G; Y=C or T; K=G or T.

Table S1. Error rate.

	454 V6-V8			MiSeq V4 Assembled			MiSeq V7-V8 Assembled			MiSeq V6-V8 R2		
	Ins.	Del.	Sub.	Ins.	Del.	Sub.	Ins.	Del.	Sub.	Ins.	Del.	Sub.
Average												
No QC	1.55833	0.77000	2.33788	0.00441	0.02501	3.02349	0.00630	0.07551	2.03523	0.00667	3.90917	4.21250
Lenient QC	1.13368	0.57950	2.15685	0.00046	0.01317	1.47008	0.00215	0.05535	1.47008	0.00000	0.09387	1.37669
Stringent QC	0.12272	0.21745	2.20531	0.00086	0.21745	2.20531	0.00086	0.04537	1.35686	0.00000	0.07796	1.13673
Std. Dev.												
No QC	0.60560	0.08239	0.54394	0.00243	0.01466	0.35334	0.00388	0.01053	0.23679	0.00402	1.43616	0.60560
Lenient QC	0.63742	0.08480	0.55671	0.00000	0.00693	0.38657	0.00192	0.01649	0.21710	0.00000	0.05858	0.63742
Stringent QC	0.06759	0.03488	0.64771	0.00031	0.00597	0.34553	0.00067	0.02006	0.16868	0.00000	0.03899	0.06759

For the lenient QC condition, sequences having more than 5 Ns, average quality score lower than 30, or more than 10 nucleotides having a quality score lower than 15 were rejected. The stringent QC condition rejected sequences that had 1 N or more; had average quality scores lower than 33; or had more than 3 nucleotides with a quality score lower than 20. Primers used for amplification where removed *in silico* before quality filtering.

Table S2. 16S reads classification from metagenomic libraries.

	**1947.2.1687 (WL01)**	**1926.6.1680 (WL02)**	**2004.3.1713 (WL07)**	**1947.3.1687 (WL11)**
**Total reads**	399,039,592	380,204,200	359,517,560	348,284,236
**Contaminants**	8,676,422	6,289,485	5,160,841	8,145,940
**Non-contaminants**	390,363,170	373,914,715	354,356,719	340,138,296
**Non-contaminants non-rRNA**	346,099,842	331,319,449	307,830,126	299,036,812
**Non-contaminants rRNA**	44,263,328	42,595,266	46,526,593	41,101,484
**Reads used for clustering and/or classification (i.e. after merging, filtering and concatenation)**	4,276,356	3,070,676	3,177,536	3,704,636
**Reads that classified at least at the kingdom level using bootstrap >= 0.50 (euk and prok)**	2,137,295	1,492,987	1,655,595	2,094,822
**Reads that classified at least at the kingdom-bacteria/archaea level using RDP threshold >= 0.50**	40,210	34,677	36,272	35,586

Table S3. Reads and OTU counts through clustering steps for *P. suwonensis.*

	454 V6-V8	MiSeq V4 Assembled	MiSeq V7-V8 Assembled	MiSeq V6-V8 Reads 2
**QCed reads**	36,770	60,769	610,836	392,270
**100% identity OTUs**	3,680	1,943	25,061	3,043
**99% identity OTUs**	1,038	368	11,072	1,792
**99% > 2**	217	68	1,451	338
**After de novo chimera removal**	217	67	1,448	337
**After reference chimera removal**	217	66	1,438	337
**97% OTUs**	2	3	10	20

Table S4. Reads and OTU counts through clustering steps for synthetic community.

	454 V6-V8	MiSeq V4 Assembled	MiSeq V7-V8 Assembled	MiSeq V6-V8 Reads 2
**QCed reads**	57,097	102,283	748,525	413,445
**100% identity OTUs**	8,928	7,166	35,185	7,670
**99% identity OTUs**	2,466	1,255	10,730	4,511
**99% > 2**	483	326	2,079	1,170
**After de novo chimera removal**	483	220	1,804	1,163
**After reference chimera removal**	443	216	1,211	1,150
**97% OTUs**	16	20	32	89

Table S5. Reads and OTU counts through clustering steps for wetlands samples.

	454 V6-V8	MiSeq V4 Assembled	MiSeq V7-V8 Assembled	MiSeq V6-V8 Reads 2
**QCed reads**	99,897	635,906	2,641,996	1,105,248
**100% identity OTUs**	59,083	305,841	1,164,152	162,273
**99% identity OTUs**	21,218	178,204	650,395	109,929
**99% > 2**	4,323	19,220	62,482	19,778
**After de novo chimera removal**	4,260	16,947	53,914	19,749
**After reference chimera removal**	4,189	16,695	49,956	19,589
**97% OTUs**	1,985	6,901	13,254	4,263
