# Supplementary material for: Primer and platform effects on 16S rRNA tag sequencing
Source: Front Microbiol. 2015 Aug 4;6:771. doi: 10.3389/fmicb.2015.00771 (PMC4523815; doi:10.3389/fmicb.2015.00771)
Supplement: Supplementary file 3 [file Additionalfile2.PDF]

Amplification primer list

V4 region

Miseq forward staggered refined primer list (96 barcodes ordered on 05_02_2012)

JGI primer_ID	Sequence
MiSeq_V4_fwd (515F)	**AATGATACGGCGACCACCGAGATCTACAC TCTTTCCCTACA GTGCCAGCMGCCGCGGTAA**

Miseq reverse staggered refined primer List (96 barcodes ordered on 05_02_2012)

JGI primer_ID	Sequence	Barcode
Miseq_St_Rev_1	CAAGCAGAAGACGGCATACGAGAT TTACCGACGAGT GTGACTGGAGTTCAGACGTGTGCTCTTCCGATCT GGACTACHVGGGTWTCTAAT	**TTACCGACGAGT**
Miseq_St_Rev_2	CAAGCAGAAGACGGCATACGAGAT ATTGGACACGCT GTGACTGGAGTTCAGACGTGTGCTCTTCCGATCT TGGACTACHVGGGTWTCTAAT	**ATTGGACACGCT**
Miseq_St_Rev_3	CAAGCAGAAGACGGCATACGAGAT TCGCATGGATAC GTGACTGGAGTTCAGACGTGTGCTCTTCCGATCT ACGGACTACHVGGGTWTCTAAT	**TCGCATGGATAC**
Miseq_St_Rev_4	CAAGCAGAAGACGGCATACGAGAT AGCGAACCTGTT GTGACTGGAGTTCAGACGTGTGCTCTTCCGATCT CTAGGACTACHVGGGTWTCTAAT	**AGCGAACCTGTT**
Miseq_St_Rev_5	CAAGCAGAAGACGGCATACGAGAT AGCTTCGACAGT GTGACTGGAGTTCAGACGTGTGCTCTTCCGATCT GGACTACHVGGGTWTCTAAT	**AGCTTCGACAGT**
Miseq_St_Rev_6	CAAGCAGAAGACGGCATACGAGAT GTCAGCCGTTAA GTGACTGGAGTTCAGACGTGTGCTCTTCCGATCT TGGACTACHVGGGTWTCTAAT	**GTCAGCCGTTAA**
Miseq_St_Rev_7	CAAGCAGAAGACGGCATACGAGAT TCCAGATAGCGT GTGACTGGAGTTCAGACGTGTGCTCTTCCGATCT ACGGACTACHVGGGTWTCTAAT	**TCCAGATAGCGT**
Miseq_St_Rev_8	CAAGCAGAAGACGGCATACGAGAT GAGAGTCCACTT GTGACTGGAGTTCAGACGTGTGCTCTTCCGATCT CTAGGACTACHVGGGTWTCTAAT	**GAGAGTCCACTT**
Miseq_St_Rev_9	CAAGCAGAAGACGGCATACGAGAT GCTCACAATGTG GTGACTGGAGTTCAGACGTGTGCTCTTCCGATCT GGACTACHVGGGTWTCTAAT	**GCTCACAATGTG**
Miseq_St_Rev_10	CAAGCAGAAGACGGCATACGAGAT TTGACGACATCG GTGACTGGAGTTCAGACGTGTGCTCTTCCGATCT TGGACTACHVGGGTWTCTAAT	**TTGACGACATCG**
Miseq_St_Rev_11	CAAGCAGAAGACGGCATACGAGAT CTTAGAACGTGC GTGACTGGAGTTCAGACGTGTGCTCTTCCGATCT ACGGACTACHVGGGTWTCTAAT	**CTTAGAACGTGC**
Miseq_St_Rev_12	CAAGCAGAAGACGGCATACGAGAT CGGTTCACATAG GTGACTGGAGTTCAGACGTGTGCTCTTCCGATCT CTAGGACTACHVGGGTWTCTAAT	**CGGTTCACATAG**
Miseq_St_Rev_13	CAAGCAGAAGACGGCATACGAGAT CGATAGGCCTTA GTGACTGGAGTTCAGACGTGTGCTCTTCCGATCT GGACTACHVGGGTWTCTAAT	**CGATAGGCCTTA**
Miseq_St_Rev_14	CAAGCAGAAGACGGCATACGAGAT GCTATATCCAGG GTGACTGGAGTTCAGACGTGTGCTCTTCCGATCT TGGACTACHVGGGTWTCTAAT	**GCTATATCCAGG**
Miseq_St_Rev_15	CAAGCAGAAGACGGCATACGAGAT GTCTTCAGCAAG GTGACTGGAGTTCAGACGTGTGCTCTTCCGATCT ACGGACTACHVGGGTWTCTAAT	**GTCTTCAGCAAG**
Miseq_St_Rev_16	CAAGCAGAAGACGGCATACGAGAT TAGACACCGTGT GTGACTGGAGTTCAGACGTGTGCTCTTCCGATCT CTAGGACTACHVGGGTWTCTAAT	**TAGACACCGTGT**
Miseq_St_Rev_17	CAAGCAGAAGACGGCATACGAGAT TCAGCTGACTAG GTGACTGGAGTTCAGACGTGTGCTCTTCCGATCT GGACTACHVGGGTWTCTAAT	**TCAGCTGACTAG**
Miseq_St_Rev_18	CAAGCAGAAGACGGCATACGAGAT TAAGTCGGCCTA GTGACTGGAGTTCAGACGTGTGCTCTTCCGATCT TGGACTACHVGGGTWTCTAAT	**TAAGTCGGCCTA**
Miseq_St_Rev_19	CAAGCAGAAGACGGCATACGAGAT GCTCCTTAGAAG GTGACTGGAGTTCAGACGTGTGCTCTTCCGATCT ACGGACTACHVGGGTWTCTAAT	**GCTCCTTAGAAG**
Miseq_St_Rev_20	CAAGCAGAAGACGGCATACGAGAT ATGGCCTGACTA GTGACTGGAGTTCAGACGTGTGCTCTTCCGATCT CTAGGACTACHVGGGTWTCTAAT	**ATGGCCTGACTA**
Miseq_St_Rev_21	CAAGCAGAAGACGGCATACGAGAT TTGCAAGTACCG GTGACTGGAGTTCAGACGTGTGCTCTTCCGATCT GGACTACHVGGGTWTCTAAT	**TTGCAAGTACCG**
Miseq_St_Rev_22	CAAGCAGAAGACGGCATACGAGAT CCTAGTAAGCTG GTGACTGGAGTTCAGACGTGTGCTCTTCCGATCT TGGACTACHVGGGTWTCTAAT	**CCTAGTAAGCTG**
Miseq_St_Rev_23	CAAGCAGAAGACGGCATACGAGAT CTAGGATCACTG GTGACTGGAGTTCAGACGTGTGCTCTTCCGATCT ACGGACTACHVGGGTWTCTAAT	**CTAGGATCACTG**
Miseq_St_Rev_24	CAAGCAGAAGACGGCATACGAGAT TATGAACGTCCG GTGACTGGAGTTCAGACGTGTGCTCTTCCGATCT CTAGGACTACHVGGGTWTCTAAT	**TATGAACGTCCG**

Miseq_St_Rev_25	CAAGCAGAAGACGGCATACGAGAT CTTGTGCGACAA GTGACTGGAGTTCAGACGTGTGCTCTTCCGATCT GGACTACHVGGGTWTCTAAT	**CTTGTGCGACAA**
Miseq_St_Rev_26	CAAGCAGAAGACGGCATACGAGAT CACGATGGTCAT GTGACTGGAGTTCAGACGTGTGCTCTTCCGATCT TGGACTACHVGGGTWTCTAAT	**CACGATGGTCAT**
Miseq_St_Rev_27	CAAGCAGAAGACGGCATACGAGAT ACGTGCCTTAGA GTGACTGGAGTTCAGACGTGTGCTCTTCCGATCT ACGGACTACHVGGGTWTCTAAT	**ACGTGCCTTAGA**
Miseq_St_Rev_28	CAAGCAGAAGACGGCATACGAGAT TGAACTAGCGTC GTGACTGGAGTTCAGACGTGTGCTCTTCCGATCT CTAGGACTACHVGGGTWTCTAAT	**TGAACTAGCGTC**
Miseq_St_Rev_29	CAAGCAGAAGACGGCATACGAGAT TATTCAGCGGAC GTGACTGGAGTTCAGACGTGTGCTCTTCCGATCT GGACTACHVGGGTWTCTAAT	**TATTCAGCGGAC**
Miseq_St_Rev_30	CAAGCAGAAGACGGCATACGAGAT TAATCGGTGCCA GTGACTGGAGTTCAGACGTGTGCTCTTCCGATCT TGGACTACHVGGGTWTCTAAT	**TAATCGGTGCCA**
Miseq_St_Rev_31	CAAGCAGAAGACGGCATACGAGAT GCGTCCATGAAT GTGACTGGAGTTCAGACGTGTGCTCTTCCGATCT ACGGACTACHVGGGTWTCTAAT	**GCGTCCATGAAT**
Miseq_St_Rev_32	CAAGCAGAAGACGGCATACGAGAT CGTAAGATGCCT GTGACTGGAGTTCAGACGTGTGCTCTTCCGATCT CTAGGACTACHVGGGTWTCTAAT	**CGTAAGATGCCT**
Miseq_St_Rev_33	CAAGCAGAAGACGGCATACGAGAT CTGTTACAGCGA GTGACTGGAGTTCAGACGTGTGCTCTTCCGATCT GGACTACHVGGGTWTCTAAT	**CTGTTACAGCGA**
Miseq_St_Rev_34	CAAGCAGAAGACGGCATACGAGAT ACGATCATCTGG GTGACTGGAGTTCAGACGTGTGCTCTTCCGATCT TGGACTACHVGGGTWTCTAAT	**ACGATCATCTGG**
Miseq_St_Rev_35	CAAGCAGAAGACGGCATACGAGAT GTAACGGCTCTA GTGACTGGAGTTCAGACGTGTGCTCTTCCGATCT ACGGACTACHVGGGTWTCTAAT	**GTAACGGCTCTA**
Miseq_St_Rev_36	CAAGCAGAAGACGGCATACGAGAT CCATGCTTAGAG GTGACTGGAGTTCAGACGTGTGCTCTTCCGATCT CTAGGACTACHVGGGTWTCTAAT	**CCATGCTTAGAG**
Miseq_St_Rev_37	CAAGCAGAAGACGGCATACGAGAT GTACGCACAGTT GTGACTGGAGTTCAGACGTGTGCTCTTCCGATCT GGACTACHVGGGTWTCTAAT	**GTACGCACAGTT**
Miseq_St_Rev_38	CAAGCAGAAGACGGCATACGAGAT TTAGAGCCATGC GTGACTGGAGTTCAGACGTGTGCTCTTCCGATCT TGGACTACHVGGGTWTCTAAT	**TTAGAGCCATGC**
Miseq_St_Rev_39	CAAGCAGAAGACGGCATACGAGAT ATAAGGTCGCCT GTGACTGGAGTTCAGACGTGTGCTCTTCCGATCT ACGGACTACHVGGGTWTCTAAT	**ATAAGGTCGCCT**
Miseq_St_Rev_40	CAAGCAGAAGACGGCATACGAGAT AGTGGCACTATC GTGACTGGAGTTCAGACGTGTGCTCTTCCGATCT CTAGGACTACHVGGGTWTCTAAT	**AGTGGCACTATC**
Miseq_St_Rev_41	CAAGCAGAAGACGGCATACGAGAT CCAGAAGTGTTC GTGACTGGAGTTCAGACGTGTGCTCTTCCGATCT GGACTACHVGGGTWTCTAAT	**CCAGAAGTGTTC**
Miseq_St_Rev_42	CAAGCAGAAGACGGCATACGAGAT CTACTAGCGGTA GTGACTGGAGTTCAGACGTGTGCTCTTCCGATCT TGGACTACHVGGGTWTCTAAT	**CTACTAGCGGTA**
Miseq_St_Rev_43	CAAGCAGAAGACGGCATACGAGAT TAGCGTTCCAGA GTGACTGGAGTTCAGACGTGTGCTCTTCCGATCT ACGGACTACHVGGGTWTCTAAT	**TAGCGTTCCAGA**
Miseq_St_Rev_44	CAAGCAGAAGACGGCATACGAGAT GTGAGTCATACC GTGACTGGAGTTCAGACGTGTGCTCTTCCGATCT CTAGGACTACHVGGGTWTCTAAT	**GTGAGTCATACC**
Miseq_St_Rev_45	CAAGCAGAAGACGGCATACGAGAT TGGTCCTACAAG GTGACTGGAGTTCAGACGTGTGCTCTTCCGATCT GGACTACHVGGGTWTCTAAT	**TGGTCCTACAAG**
Miseq_St_Rev_46	CAAGCAGAAGACGGCATACGAGAT TACGCGTACAGT GTGACTGGAGTTCAGACGTGTGCTCTTCCGATCT TGGACTACHVGGGTWTCTAAT	**TACGCGTACAGT**
Miseq_St_Rev_47	CAAGCAGAAGACGGCATACGAGAT GAGCCATCTGTA GTGACTGGAGTTCAGACGTGTGCTCTTCCGATCT ACGGACTACHVGGGTWTCTAAT	**GAGCCATCTGTA**
Miseq_St_Rev_48	CAAGCAGAAGACGGCATACGAGAT CGTCCGTATGAA GTGACTGGAGTTCAGACGTGTGCTCTTCCGATCT CTAGGACTACHVGGGTWTCTAAT	**CGTCCGTATGAA**
Miseq_St_Rev_49	CAAGCAGAAGACGGCATACGAGAT GATACGTTCGCA GTGACTGGAGTTCAGACGTGTGCTCTTCCGATCT GGACTACHVGGGTWTCTAAT	**GATACGTTCGCA**
Miseq_St_Rev_50	CAAGCAGAAGACGGCATACGAGAT CAGCTGGTTCAA GTGACTGGAGTTCAGACGTGTGCTCTTCCGATCT TGGACTACHVGGGTWTCTAAT	**CAGCTGGTTCAA**
Miseq_St_Rev_51	CAAGCAGAAGACGGCATACGAGAT TTAAGCGCCTGA GTGACTGGAGTTCAGACGTGTGCTCTTCCGATCT ACGGACTACHVGGGTWTCTAAT	**TTAAGCGCCTGA**
Miseq_St_Rev_52	CAAGCAGAAGACGGCATACGAGAT CCTGCGAAGTAT GTGACTGGAGTTCAGACGTGTGCTCTTCCGATCT CTAGGACTACHVGGGTWTCTAAT	**CCTGCGAAGTAT**
Miseq_St_Rev_53	CAAGCAGAAGACGGCATACGAGAT TTGTAGCCGACA GTGACTGGAGTTCAGACGTGTGCTCTTCCGATCT GGACTACHVGGGTWTCTAAT	**TTGTAGCCGACA**
Miseq_St_Rev_54	CAAGCAGAAGACGGCATACGAGAT TCTGTAGAGCCA GTGACTGGAGTTCAGACGTGTGCTCTTCCGATCT TGGACTACHVGGGTWTCTAAT	**TCTGTAGAGCCA**
Miseq_St_Rev_55	CAAGCAGAAGACGGCATACGAGAT CTATTAAGCGGC GTGACTGGAGTTCAGACGTGTGCTCTTCCGATCT ACGGACTACHVGGGTWTCTAAT	**CTATTAAGCGGC**
Miseq_St_Rev_56	CAAGCAGAAGACGGCATACGAGAT CTCTGAGGTAAC GTGACTGGAGTTCAGACGTGTGCTCTTCCGATCT CTAGGACTACHVGGGTWTCTAAT	**CTCTGAGGTAAC**
Miseq_St_Rev_57	CAAGCAGAAGACGGCATACGAGAT CAGGATTCGTAC GTGACTGGAGTTCAGACGTGTGCTCTTCCGATCT GGACTACHVGGGTWTCTAAT	**CAGGATTCGTAC**
Miseq_St_Rev_58	CAAGCAGAAGACGGCATACGAGAT TCACTGCTAGGA GTGACTGGAGTTCAGACGTGTGCTCTTCCGATCT TGGACTACHVGGGTWTCTAAT	**TCACTGCTAGGA**
Miseq_St_Rev_59	CAAGCAGAAGACGGCATACGAGAT ACATGTCACGTG GTGACTGGAGTTCAGACGTGTGCTCTTCCGATCT ACGGACTACHVGGGTWTCTAAT	**ACATGTCACGTG**
Miseq_St_Rev_60	CAAGCAGAAGACGGCATACGAGAT ATTCTGCCGAAG GTGACTGGAGTTCAGACGTGTGCTCTTCCGATCT CTAGGACTACHVGGGTWTCTAAT	**ATTCTGCCGAAG**

Miseq_St_Rev_61	CAAGCAGAAGACGGCATACGAGAT TACACGCTGATG GTGACTGGAGTTCAGACGTGTGCTCTTCCGATCT GGACTACHVGGGTWTCTAAT	TACACGCTGATG
Miseq_St_Rev_62	CAAGCAGAAGACGGCATACGAGAT TGCATACACTGG GTGACTGGAGTTCAGACGTGTGCTCTTCCGATCT TGGACTACHVGGGTWTCTAAT	TGCATACACTGG
Miseq_St_Rev_63	CAAGCAGAAGACGGCATACGAGAT ACGCAATGTCTG GTGACTGGAGTTCAGACGTGTGCTCTTCCGATCT ACGGACTACHVGGGTWTCTAAT	ACGCAATGTCTG
Miseq_St_Rev_64	CAAGCAGAAGACGGCATACGAGAT GCTCGAAGATTC GTGACTGGAGTTCAGACGTGTGCTCTTCCGATCT CTAGGACTACHVGGGTWTCTAAT	GCTCGAAGATTC
Miseq_St_Rev_65	CAAGCAGAAGACGGCATACGAGAT AGACGTTGCTAC GTGACTGGAGTTCAGACGTGTGCTCTTCCGATCT GGACTACHVGGGTWTCTAAT	AGACGTTGCTAC
Miseq_St_Rev_66	CAAGCAGAAGACGGCATACGAGAT TAGAGCTGCCAT GTGACTGGAGTTCAGACGTGTGCTCTTCCGATCT TGGACTACHVGGGTWTCTAAT	TAGAGCTGCCAT
Miseq_St_Rev_67	CAAGCAGAAGACGGCATACGAGAT GGTAACCTCTGA GTGACTGGAGTTCAGACGTGTGCTCTTCCGATCT ACGGACTACHVGGGTWTCTAAT	GGTAACCTCTGA
Miseq_St_Rev_68	CAAGCAGAAGACGGCATACGAGAT GACTTCATGCGA GTGACTGGAGTTCAGACGTGTGCTCTTCCGATCT CTAGGACTACHVGGGTWTCTAAT	GACTTCATGCGA
Miseq_St_Rev_69	CAAGCAGAAGACGGCATACGAGAT CTGCATACTGAG GTGACTGGAGTTCAGACGTGTGCTCTTCCGATCT GGACTACHVGGGTWTCTAAT	CTGCATACTGAG
Miseq_St_Rev_70	CAAGCAGAAGACGGCATACGAGAT TAAGGCATCGCT GTGACTGGAGTTCAGACGTGTGCTCTTCCGATCT TGGACTACHVGGGTWTCTAAT	TAAGGCATCGCT
Miseq_St_Rev_71	CAAGCAGAAGACGGCATACGAGAT AGTATTCGCGCA GTGACTGGAGTTCAGACGTGTGCTCTTCCGATCT ACGGACTACHVGGGTWTCTAAT	AGTATTCGCGCA
Miseq_St_Rev_72	CAAGCAGAAGACGGCATACGAGAT TTCGCAGATACG GTGACTGGAGTTCAGACGTGTGCTCTTCCGATCT CTAGGACTACHVGGGTWTCTAAT	TTCGCAGATACG
Miseq_St_Rev_73	CAAGCAGAAGACGGCATACGAGAT GCACCTGTTGAA GTGACTGGAGTTCAGACGTGTGCTCTTCCGATCT GGACTACHVGGGTWTCTAAT	GCACCTGTTGAA
Miseq_St_Rev_74	CAAGCAGAAGACGGCATACGAGAT CTCATGGTAGCA GTGACTGGAGTTCAGACGTGTGCTCTTCCGATCT TGGACTACHVGGGTWTCTAAT	CTCATGGTAGCA
Miseq_St_Rev_75	CAAGCAGAAGACGGCATACGAGAT ACTAGTTGGACC GTGACTGGAGTTCAGACGTGTGCTCTTCCGATCT ACGGACTACHVGGGTWTCTAAT	ACTAGTTGGACC
Miseq_St_Rev_76	CAAGCAGAAGACGGCATACGAGAT GCGGACTATTCA GTGACTGGAGTTCAGACGTGTGCTCTTCCGATCT CTAGGACTACHVGGGTWTCTAAT	GCGGACTATTCA
Miseq_St_Rev_77	CAAGCAGAAGACGGCATACGAGAT ATCGCTTAAGGC GTGACTGGAGTTCAGACGTGTGCTCTTCCGATCT GGACTACHVGGGTWTCTAAT	ATCGCTTAAGGC
Miseq_St_Rev_78	CAAGCAGAAGACGGCATACGAGAT TCAGGACGTATC GTGACTGGAGTTCAGACGTGTGCTCTTCCGATCT TGGACTACHVGGGTWTCTAAT	TCAGGACGTATC
Miseq_St_Rev_79	CAAGCAGAAGACGGCATACGAGAT GCATTACTGGAC GTGACTGGAGTTCAGACGTGTGCTCTTCCGATCT ACGGACTACHVGGGTWTCTAAT	GCATTACTGGAC
Miseq_St_Rev_80	CAAGCAGAAGACGGCATACGAGAT GCTATGGAACTC GTGACTGGAGTTCAGACGTGTGCTCTTCCGATCT CTAGGACTACHVGGGTWTCTAAT	GCTATGGAACTC
Miseq_St_Rev_81	CAAGCAGAAGACGGCATACGAGAT GATTGTGCAACC GTGACTGGAGTTCAGACGTGTGCTCTTCCGATCT GGACTACHVGGGTWTCTAAT	GATTGTGCAACC
Miseq_St_Rev_82	CAAGCAGAAGACGGCATACGAGAT AGCCTCATGATG GTGACTGGAGTTCAGACGTGTGCTCTTCCGATCT TGGACTACHVGGGTWTCTAAT	AGCCTCATGATG
Miseq_St_Rev_83	CAAGCAGAAGACGGCATACGAGAT AACTCCTGTGGA GTGACTGGAGTTCAGACGTGTGCTCTTCCGATCT ACGGACTACHVGGGTWTCTAAT	AACTCCTGTGGA
Miseq_St_Rev_84	CAAGCAGAAGACGGCATACGAGAT TAGAAGGCTCCT GTGACTGGAGTTCAGACGTGTGCTCTTCCGATCT CTAGGACTACHVGGGTWTCTAAT	TAGAAGGCTCCT
Miseq_St_Rev_85	CAAGCAGAAGACGGCATACGAGAT GACTAGTCAGCT GTGACTGGAGTTCAGACGTGTGCTCTTCCGATCT GGACTACHVGGGTWTCTAAT	GACTAGTCAGCT
Miseq_St_Rev_86	CAAGCAGAAGACGGCATACGAGAT GGATACTCGCAT GTGACTGGAGTTCAGACGTGTGCTCTTCCGATCT TGGACTACHVGGGTWTCTAAT	GGATACTCGCAT
Miseq_St_Rev_87	CAAGCAGAAGACGGCATACGAGAT CCGACATTGTAG GTGACTGGAGTTCAGACGTGTGCTCTTCCGATCT ACGGACTACHVGGGTWTCTAAT	CCGACATTGTAG
Miseq_St_Rev_88	CAAGCAGAAGACGGCATACGAGAT TCGTGACGCTAA GTGACTGGAGTTCAGACGTGTGCTCTTCCGATCT CTAGGACTACHVGGGTWTCTAAT	TCGTGACGCTAA
Miseq_St_Rev_89	CAAGCAGAAGACGGCATACGAGAT GGCCTATAAGTC GTGACTGGAGTTCAGACGTGTGCTCTTCCGATCT GGACTACHVGGGTWTCTAAT	GGCCTATAAGTC
Miseq_St_Rev_90	CAAGCAGAAGACGGCATACGAGAT GTAGCACTCATG GTGACTGGAGTTCAGACGTGTGCTCTTCCGATCT TGGACTACHVGGGTWTCTAAT	GTAGCACTCATG
Miseq_St_Rev_91	CAAGCAGAAGACGGCATACGAGAT CTAAGACGTCGT GTGACTGGAGTTCAGACGTGTGCTCTTCCGATCT ACGGACTACHVGGGTWTCTAAT	CTAAGACGTCGT
Miseq_St_Rev_92	CAAGCAGAAGACGGCATACGAGAT CGTGCACAATTG GTGACTGGAGTTCAGACGTGTGCTCTTCCGATCT CTAGGACTACHVGGGTWTCTAAT	CGTGCACAATTG
Miseq_St_Rev_93	CAAGCAGAAGACGGCATACGAGAT TGTAACGCCGAT GTGACTGGAGTTCAGACGTGTGCTCTTCCGATCT GGACTACHVGGGTWTCTAAT	TGTAACGCCGAT
Miseq_St_Rev_94	CAAGCAGAAGACGGCATACGAGAT ATGCGAGACTTC GTGACTGGAGTTCAGACGTGTGCTCTTCCGATCT TGGACTACHVGGGTWTCTAAT	ATGCGAGACTTC
Miseq_St_Rev_95	CAAGCAGAAGACGGCATACGAGAT CCGTCAAGATGT GTGACTGGAGTTCAGACGTGTGCTCTTCCGATCT ACGGACTACHVGGGTWTCTAAT	CCGTCAAGATGT
Miseq_St_Rev_96	CAAGCAGAAGACGGCATACGAGAT TAGTAGCACCTG GTGACTGGAGTTCAGACGTGTGCTCTTCCGATCT CTAGGACTACHVGGGTWTCTAAT	TAGTAGCACCTG

V7-V8 region

Miseq reverse staggered refined primer list	
**JGI primer_ID**	**Sequence**
MiSeq_V7_fwd (1114F)	AATGATACGGCGACCACCGAGATCTACAC TCTTTCCCTACA GCAACGAGCGCAACCC

Miseq reverse staggered refined primer list

JGI primer_ID	Sequence	Barcode
Miseq_V8_Rev_1	CAAGCAGAAGACGGCATACGAGAT TTACCGACGAGT GTGACTGGAGTTCAGACGTGTGCTCTTCCGATCT ACGGGCGGTGTGTRC	TTACCGACGAGT
Miseq_V8_Rev_2	CAAGCAGAAGACGGCATACGAGAT ATTGGACACGCT GTGACTGGAGTTCAGACGTGTGCTCTTCCGATCT GACGGGCGGTGTGTRC	ATTGGACACGCT
Miseq_V8_Rev_3	CAAGCAGAAGACGGCATACGAGAT TCGCATGGATAC GTGACTGGAGTTCAGACGTGTGCTCTTCCGATCT TGACGGGCGGTGTGTRC	TCGCATGGATAC
Miseq_V8_Rev_4	CAAGCAGAAGACGGCATACGAGAT AGCGAACCTGTT GTGACTGGAGTTCAGACGTGTGCTCTTCCGATCT CTGACGGGCGGTGTGTRC	AGCGAACCTGTT
Miseq_V8_Rev_5	CAAGCAGAAGACGGCATACGAGAT AGCTTCGACAGT GTGACTGGAGTTCAGACGTGTGCTCTTCCGATCT ACGGGCGGTGTGTRC	AGCTTCGACAGT
Miseq_V8_Rev_6	CAAGCAGAAGACGGCATACGAGAT GTCAGCCGTTAA GTGACTGGAGTTCAGACGTGTGCTCTTCCGATCT GACGGGCGGTGTGTRC	GTCAGCCGTTAA
Miseq_V8_Rev_7	CAAGCAGAAGACGGCATACGAGAT TCCAGATAGCGT GTGACTGGAGTTCAGACGTGTGCTCTTCCGATCT TGACGGGCGGTGTGTRC	TCCAGATAGCGT
Miseq_V8_Rev_8	CAAGCAGAAGACGGCATACGAGAT GAGAGTCCACTT GTGACTGGAGTTCAGACGTGTGCTCTTCCGATCT CTGACGGGCGGTGTGTRC	GAGAGTCCACTT
Miseq_V8_Rev_9	CAAGCAGAAGACGGCATACGAGAT GCTCACAATGTG GTGACTGGAGTTCAGACGTGTGCTCTTCCGATCT ACGGGCGGTGTGTRC	GCTCACAATGTG
Miseq_V8_Rev_10	CAAGCAGAAGACGGCATACGAGAT TTGACGACATCG GTGACTGGAGTTCAGACGTGTGCTCTTCCGATCT GACGGGCGGTGTGTRC	TTGACGACATCG
Miseq_V8_Rev_11	CAAGCAGAAGACGGCATACGAGAT CTTAGAACGTGC GTGACTGGAGTTCAGACGTGTGCTCTTCCGATCT TGACGGGCGGTGTGTRC	CTTAGAACGTGC
Miseq_V8_Rev_12	CAAGCAGAAGACGGCATACGAGAT CGGTTCACATAG GTGACTGGAGTTCAGACGTGTGCTCTTCCGATCT CTGACGGGCGGTGTGTRC	CGGTTCACATAG
Miseq_V8_Rev_13	CAAGCAGAAGACGGCATACGAGAT CGATAGGCCTTA GTGACTGGAGTTCAGACGTGTGCTCTTCCGATCT ACGGGCGGTGTGTRC	CGATAGGCCTTA
Miseq_V8_Rev_14	CAAGCAGAAGACGGCATACGAGAT GCTATATCCAGG GTGACTGGAGTTCAGACGTGTGCTCTTCCGATCT GACGGGCGGTGTGTRC	GCTATATCCAGG
Miseq_V8_Rev_15	CAAGCAGAAGACGGCATACGAGAT GTCTTCAGCAAG GTGACTGGAGTTCAGACGTGTGCTCTTCCGATCT TGACGGGCGGTGTGTRC	GTCTTCAGCAAG
Miseq_V8_Rev_16	CAAGCAGAAGACGGCATACGAGAT TAGACACCGTGT GTGACTGGAGTTCAGACGTGTGCTCTTCCGATCT CTGACGGGCGGTGTGTRC	TAGACACCGTGT
Miseq_V8_Rev_17	CAAGCAGAAGACGGCATACGAGAT TCAGCTGACTAG GTGACTGGAGTTCAGACGTGTGCTCTTCCGATCT ACGGGCGGTGTGTRC	TCAGCTGACTAG
Miseq_V8_Rev_18	CAAGCAGAAGACGGCATACGAGAT TAAGTCGGCCTA GTGACTGGAGTTCAGACGTGTGCTCTTCCGATCT GACGGGCGGTGTGTRC	TAAGTCGGCCTA
Miseq_V8_Rev_19	CAAGCAGAAGACGGCATACGAGAT GCTCCTTAGAAG GTGACTGGAGTTCAGACGTGTGCTCTTCCGATCT TGACGGGCGGTGTGTRC	GCTCCTTAGAAG
Miseq_V8_Rev_20	CAAGCAGAAGACGGCATACGAGAT GCTCCTTAGAAG GTGACTGGAGTTCAGACGTGTGCTCTTCCGATCT CTGACGGGCGGTGTGTRC	ATGGCCTGACTA
Miseq_V8_Rev_21	CAAGCAGAAGACGGCATACGAGAT TTGCAAGTACCG GTGACTGGAGTTCAGACGTGTGCTCTTCCGATCT ACGGGCGGTGTGTRC	TTGCAAGTACCG
Miseq_V8_Rev_22	CAAGCAGAAGACGGCATACGAGAT CCTAGTAAGCTG GTGACTGGAGTTCAGACGTGTGCTCTTCCGATCT GACGGGCGGTGTGTRC	CCTAGTAAGCTG
Miseq_V8_Rev_23	CAAGCAGAAGACGGCATACGAGAT CTAGGATCACTG GTGACTGGAGTTCAGACGTGTGCTCTTCCGATCT TGACGGGCGGTGTGTRC	CTAGGATCACTG
Miseq_V8_Rev_24	CAAGCAGAAGACGGCATACGAGAT TATGAACGTCCG GTGACTGGAGTTCAGACGTGTGCTCTTCCGATCT CTGACGGGCGGTGTGTRC	TATGAACGTCCG
Miseq_V8_Rev_25	CAAGCAGAAGACGGCATACGAGAT CTTGTGCGACAA GTGACTGGAGTTCAGACGTGTGCTCTTCCGATCT ACGGGCGGTGTGTRC	CTTGTGCGACAA
Miseq_V8_Rev_26	CAAGCAGAAGACGGCATACGAGAT CACGATGGTCAT GTGACTGGAGTTCAGACGTGTGCTCTTCCGATCT GACGGGCGGTGTGTRC	CACGATGGTCAT
Miseq_V8_Rev_27	CAAGCAGAAGACGGCATACGAGAT ACGTGCCTTAGA GTGACTGGAGTTCAGACGTGTGCTCTTCCGATCT TGACGGGCGGTGTGTRC	ACGTGCCTTAGA
Miseq_V8_Rev_28	CAAGCAGAAGACGGCATACGAGAT TGAACTAGCGTC GTGACTGGAGTTCAGACGTGTGCTCTTCCGATCT CTGACGGGCGGTGTGTRC	TGAACTAGCGTC

Miseq_V8_Rev_29	CAAGCAGAAGACGGCATACGAGAT TATTCAGCGGAC GTGACTGGAGTTCAGACGTGTGCTCTTCCGATCT ACGGGCGGTGTGTRC	TATTCAGCGGAC
Miseq_V8_Rev_30	CAAGCAGAAGACGGCATACGAGAT TAATCGGTGCCA GTGACTGGAGTTCAGACGTGTGCTCTTCCGATCT GACGGGCGGTGTGTRC	TAATCGGTGCCA
Miseq_V8_Rev_31	CAAGCAGAAGACGGCATACGAGAT GCGTCCATGAAT GTGACTGGAGTTCAGACGTGTGCTCTTCCGATCT TGACGGGCGGTGTGTRC	GCGTCCATGAAT
Miseq_V8_Rev_32	CAAGCAGAAGACGGCATACGAGAT CGTAAGATGCCT GTGACTGGAGTTCAGACGTGTGCTCTTCCGATCT CTGACGGGCGGTGTGTRC	CGTAAGATGCCT
Miseq_V8_Rev_33	CAAGCAGAAGACGGCATACGAGAT CTGTTACAGCGA GTGACTGGAGTTCAGACGTGTGCTCTTCCGATCT ACGGGCGGTGTGTRC	CTGTTACAGCGA
Miseq_V8_Rev_34	CAAGCAGAAGACGGCATACGAGAT ACGATCATCTGG GTGACTGGAGTTCAGACGTGTGCTCTTCCGATCT GACGGGCGGTGTGTRC	ACGATCATCTGG
Miseq_V8_Rev_35	CAAGCAGAAGACGGCATACGAGAT GTAACGGCTCTA GTGACTGGAGTTCAGACGTGTGCTCTTCCGATCT TGACGGGCGGTGTGTRC	GTAACGGCTCTA
Miseq_V8_Rev_36	CAAGCAGAAGACGGCATACGAGAT CCATGCTTAGAG GTGACTGGAGTTCAGACGTGTGCTCTTCCGATCT CTGACGGGCGGTGTGTRC	CCATGCTTAGAG
Miseq_V8_Rev_37	CAAGCAGAAGACGGCATACGAGAT GTACGCACAGTT GTGACTGGAGTTCAGACGTGTGCTCTTCCGATCT ACGGGCGGTGTGTRC	GTACGCACAGTT
Miseq_V8_Rev_38	CAAGCAGAAGACGGCATACGAGAT TTAGAGCCATGC GTGACTGGAGTTCAGACGTGTGCTCTTCCGATCT GACGGGCGGTGTGTRC	TTAGAGCCATGC
Miseq_V8_Rev_39	CAAGCAGAAGACGGCATACGAGAT ATAAGGTCGCCT GTGACTGGAGTTCAGACGTGTGCTCTTCCGATCT TGACGGGCGGTGTGTRC	ATAAGGTCGCCT
Miseq_V8_Rev_40	CAAGCAGAAGACGGCATACGAGAT AGTGGCACTATC GTGACTGGAGTTCAGACGTGTGCTCTTCCGATCT CTGACGGGCGGTGTGTRC	AGTGGCACTATC
Miseq_V8_Rev_41	CAAGCAGAAGACGGCATACGAGAT CCAGAAGTGTTC GTGACTGGAGTTCAGACGTGTGCTCTTCCGATCT ACGGGCGGTGTGTRC	CCAGAAGTGTTC
Miseq_V8_Rev_42	CAAGCAGAAGACGGCATACGAGAT CTACTAGCGGTA GTGACTGGAGTTCAGACGTGTGCTCTTCCGATCT GACGGGCGGTGTGTRC	CTACTAGCGGTA
Miseq_V8_Rev_43	CAAGCAGAAGACGGCATACGAGAT TAGCGTTCCAGA GTGACTGGAGTTCAGACGTGTGCTCTTCCGATCT TGACGGGCGGTGTGTRC	TAGCGTTCCAGA
Miseq_V8_Rev_44	CAAGCAGAAGACGGCATACGAGAT GTGAGTCATACC GTGACTGGAGTTCAGACGTGTGCTCTTCCGATCT CTGACGGGCGGTGTGTRC	GTGAGTCATACC
Miseq_V8_Rev_45	CAAGCAGAAGACGGCATACGAGAT TGGTCCTACAAG GTGACTGGAGTTCAGACGTGTGCTCTTCCGATCT ACGGGCGGTGTGTRC	TGGTCCTACAAG
Miseq_V8_Rev_46	CAAGCAGAAGACGGCATACGAGAT TACGCGTACAGT GTGACTGGAGTTCAGACGTGTGCTCTTCCGATCT GACGGGCGGTGTGTRC	TACGCGTACAGT
Miseq_V8_Rev_47	CAAGCAGAAGACGGCATACGAGAT GAGCCATCTGTA GTGACTGGAGTTCAGACGTGTGCTCTTCCGATCT TGACGGGCGGTGTGTRC	GAGCCATCTGTA
Miseq_V8_Rev_48	CAAGCAGAAGACGGCATACGAGAT CGTCCGTATGAA GTGACTGGAGTTCAGACGTGTGCTCTTCCGATCT CTGACGGGCGGTGTGTRC	CGTCCGTATGAA
Miseq_V8_Rev_49	CAAGCAGAAGACGGCATACGAGAT GATACGTTCGCA GTGACTGGAGTTCAGACGTGTGCTCTTCCGATCT ACGGGCGGTGTGTRC	GATACGTTCGCA
Miseq_V8_Rev_50	CAAGCAGAAGACGGCATACGAGAT CAGCTGGTTCAA GTGACTGGAGTTCAGACGTGTGCTCTTCCGATCT GACGGGCGGTGTGTRC	CAGCTGGTTCAA
Miseq_V8_Rev_51	CAAGCAGAAGACGGCATACGAGAT TTAAGCGCCTGA GTGACTGGAGTTCAGACGTGTGCTCTTCCGATCT TGACGGGCGGTGTGTRC	TTAAGCGCCTGA
Miseq_V8_Rev_52	CAAGCAGAAGACGGCATACGAGAT CCTGCGAAGTAT GTGACTGGAGTTCAGACGTGTGCTCTTCCGATCT CTGACGGGCGGTGTGTRC	CCTGCGAAGTAT
Miseq_V8_Rev_53	CAAGCAGAAGACGGCATACGAGAT TTGTAGCCGACA GTGACTGGAGTTCAGACGTGTGCTCTTCCGATCT ACGGGCGGTGTGTRC	TTGTAGCCGACA
Miseq_V8_Rev_54	CAAGCAGAAGACGGCATACGAGAT TCTGTAGAGCCA GTGACTGGAGTTCAGACGTGTGCTCTTCCGATCT GACGGGCGGTGTGTRC	TCTGTAGAGCCA
Miseq_V8_Rev_55	CAAGCAGAAGACGGCATACGAGAT CTATTAAGCGGC GTGACTGGAGTTCAGACGTGTGCTCTTCCGATCT TGACGGGCGGTGTGTRC	CTATTAAGCGGC
Miseq_V8_Rev_56	CAAGCAGAAGACGGCATACGAGAT CTCTGAGGTAAC GTGACTGGAGTTCAGACGTGTGCTCTTCCGATCT CTGACGGGCGGTGTGTRC	CTCTGAGGTAAC
Miseq_V8_Rev_57	CAAGCAGAAGACGGCATACGAGAT CAGGATTCGTAC GTGACTGGAGTTCAGACGTGTGCTCTTCCGATCT ACGGGCGGTGTGTRC	CAGGATTCGTAC
Miseq_V8_Rev_58	CAAGCAGAAGACGGCATACGAGAT TCACTGCTAGGA GTGACTGGAGTTCAGACGTGTGCTCTTCCGATCT GACGGGCGGTGTGTRC	TCACTGCTAGGA
Miseq_V8_Rev_59	CAAGCAGAAGACGGCATACGAGAT ACATGTCACGTG GTGACTGGAGTTCAGACGTGTGCTCTTCCGATCT TGACGGGCGGTGTGTRC	ACATGTCACGTG
Miseq_V8_Rev_60	CAAGCAGAAGACGGCATACGAGAT ATTCTGCCGAAG GTGACTGGAGTTCAGACGTGTGCTCTTCCGATCT CTGACGGGCGGTGTGTRC	ATTCTGCCGAAG
Miseq_V8_Rev_61	CAAGCAGAAGACGGCATACGAGAT TACACGCTGATG GTGACTGGAGTTCAGACGTGTGCTCTTCCGATCT ACGGGCGGTGTGTRC	TACACGCTGATG
Miseq_V8_Rev_62	CAAGCAGAAGACGGCATACGAGAT TGCATACACTGG GTGACTGGAGTTCAGACGTGTGCTCTTCCGATCT GACGGGCGGTGTGTRC	TGCATACACTGG
Miseq_V8_Rev_63	CAAGCAGAAGACGGCATACGAGAT ACGCAATGTCTG GTGACTGGAGTTCAGACGTGTGCTCTTCCGATCT TGACGGGCGGTGTGTRC	ACGCAATGTCTG
Miseq_V8_Rev_64	CAAGCAGAAGACGGCATACGAGAT GCTCGAAGATTC GTGACTGGAGTTCAGACGTGTGCTCTTCCGATCT CTGACGGGCGGTGTGTRC	GCTCGAAGATTC

Miseq_V8_Rev_65	CAAGCAGAAGACGGCATACGAGAT AGACGTTGCTAC GTGACTGGAGTTCAGACGTGTGCTCTTCCGATCT ACGGGCGGTGTGTRC	AGACGTTGCTAC
Miseq_V8_Rev_66	CAAGCAGAAGACGGCATACGAGAT TAGAGCTGCCAT GTGACTGGAGTTCAGACGTGTGCTCTTCCGATCT GACGGGCGGTGTGTRC	TAGAGCTGCCAT
Miseq_V8_Rev_67	CAAGCAGAAGACGGCATACGAGAT GGTAACCTCTGA GTGACTGGAGTTCAGACGTGTGCTCTTCCGATCT TGACGGGCGGTGTGTRC	GGTAACCTCTGA
Miseq_V8_Rev_68	CAAGCAGAAGACGGCATACGAGAT GACTTCATGCGA GTGACTGGAGTTCAGACGTGTGCTCTTCCGATCT CTGACGGGCGGTGTGTRC	GACTTCATGCGA
Miseq_V8_Rev_69	CAAGCAGAAGACGGCATACGAGAT CTGCATACTGAG GTGACTGGAGTTCAGACGTGTGCTCTTCCGATCT ACGGGCGGTGTGTRC	CTGCATACTGAG
Miseq_V8_Rev_70	CAAGCAGAAGACGGCATACGAGAT TAAGGCATCGCT GTGACTGGAGTTCAGACGTGTGCTCTTCCGATCT GACGGGCGGTGTGTRC	TAAGGCATCGCT
Miseq_V8_Rev_71	CAAGCAGAAGACGGCATACGAGAT AGTATTCGCGCA GTGACTGGAGTTCAGACGTGTGCTCTTCCGATCT TGACGGGCGGTGTGTRC	AGTATTCGCGCA
Miseq_V8_Rev_72	CAAGCAGAAGACGGCATACGAGAT TTCGCAGATACG GTGACTGGAGTTCAGACGTGTGCTCTTCCGATCT CTGACGGGCGGTGTGTRC	TTCGCAGATACG
Miseq_V8_Rev_73	CAAGCAGAAGACGGCATACGAGAT GCACCTGTTGAA GTGACTGGAGTTCAGACGTGTGCTCTTCCGATCT ACGGGCGGTGTGTRC	GCACCTGTTGAA
Miseq_V8_Rev_74	CAAGCAGAAGACGGCATACGAGAT CTCATGGTAGCA GTGACTGGAGTTCAGACGTGTGCTCTTCCGATCT GACGGGCGGTGTGTRC	CTCATGGTAGCA
Miseq_V8_Rev_75	CAAGCAGAAGACGGCATACGAGAT ACTAGTTGGACC GTGACTGGAGTTCAGACGTGTGCTCTTCCGATCT TGACGGGCGGTGTGTRC	ACTAGTTGGACC
Miseq_V8_Rev_76	CAAGCAGAAGACGGCATACGAGAT GCGGACTATTCA GTGACTGGAGTTCAGACGTGTGCTCTTCCGATCT CTGACGGGCGGTGTGTRC	GCGGACTATTCA
Miseq_V8_Rev_77	CAAGCAGAAGACGGCATACGAGAT ATCGCTTAAGGC GTGACTGGAGTTCAGACGTGTGCTCTTCCGATCT ACGGGCGGTGTGTRC	ATCGCTTAAGGC
Miseq_V8_Rev_78	CAAGCAGAAGACGGCATACGAGAT TCAGGACGTATC GTGACTGGAGTTCAGACGTGTGCTCTTCCGATCT GACGGGCGGTGTGTRC	TCAGGACGTATC
Miseq_V8_Rev_79	CAAGCAGAAGACGGCATACGAGAT GCATTACTGGAC GTGACTGGAGTTCAGACGTGTGCTCTTCCGATCT TGACGGGCGGTGTGTRC	GCATTACTGGAC
Miseq_V8_Rev_80	CAAGCAGAAGACGGCATACGAGAT GCTATGGAACTC GTGACTGGAGTTCAGACGTGTGCTCTTCCGATCT CTGACGGGCGGTGTGTRC	GCTATGGAACTC
Miseq_V8_Rev_81	CAAGCAGAAGACGGCATACGAGAT GATTGTGCAACC GTGACTGGAGTTCAGACGTGTGCTCTTCCGATCT ACGGGCGGTGTGTRC	GATTGTGCAACC
Miseq_V8_Rev_82	CAAGCAGAAGACGGCATACGAGAT AGCCTCATGATG GTGACTGGAGTTCAGACGTGTGCTCTTCCGATCT GACGGGCGGTGTGTRC	AGCCTCATGATG
Miseq_V8_Rev_83	CAAGCAGAAGACGGCATACGAGAT AACTCCTGTGGA GTGACTGGAGTTCAGACGTGTGCTCTTCCGATCT TGACGGGCGGTGTGTRC	AACTCCTGTGGA
Miseq_V8_Rev_84	CAAGCAGAAGACGGCATACGAGAT TAGAAGGCTCCT GTGACTGGAGTTCAGACGTGTGCTCTTCCGATCT CTGACGGGCGGTGTGTRC	TAGAAGGCTCCT
Miseq_V8_Rev_85	CAAGCAGAAGACGGCATACGAGAT GACTAGTCAGCT GTGACTGGAGTTCAGACGTGTGCTCTTCCGATCT ACGGGCGGTGTGTRC	GACTAGTCAGCT
Miseq_V8_Rev_86	CAAGCAGAAGACGGCATACGAGAT GGATACTCGCAT GTGACTGGAGTTCAGACGTGTGCTCTTCCGATCT GACGGGCGGTGTGTRC	GGATACTCGCAT
Miseq_V8_Rev_87	CAAGCAGAAGACGGCATACGAGAT CCGACATTGTAG GTGACTGGAGTTCAGACGTGTGCTCTTCCGATCT TGACGGGCGGTGTGTRC	CCGACATTGTAG
Miseq_V8_Rev_88	CAAGCAGAAGACGGCATACGAGAT TCGTGACGCTAA GTGACTGGAGTTCAGACGTGTGCTCTTCCGATCT CTGACGGGCGGTGTGTRC	TCGTGACGCTAA
Miseq_V8_Rev_89	CAAGCAGAAGACGGCATACGAGAT GGCCTATAAGTC GTGACTGGAGTTCAGACGTGTGCTCTTCCGATCT ACGGGCGGTGTGTRC	GGCCTATAAGTC
Miseq_V8_Rev_90	CAAGCAGAAGACGGCATACGAGAT GTAGCACTCATG GTGACTGGAGTTCAGACGTGTGCTCTTCCGATCT GACGGGCGGTGTGTRC	GTAGCACTCATG
Miseq_V8_Rev_91	CAAGCAGAAGACGGCATACGAGAT CTAAGACGTCGT GTGACTGGAGTTCAGACGTGTGCTCTTCCGATCT TGACGGGCGGTGTGTRC	CTAAGACGTCGT
Miseq_V8_Rev_92	CAAGCAGAAGACGGCATACGAGAT CGTGCACAATTG GTGACTGGAGTTCAGACGTGTGCTCTTCCGATCT CTGACGGGCGGTGTGTRC	CGTGCACAATTG
Miseq_V8_Rev_93	CAAGCAGAAGACGGCATACGAGAT TGTAACGCCGAT GTGACTGGAGTTCAGACGTGTGCTCTTCCGATCT ACGGGCGGTGTGTRC	TGTAACGCCGAT
Miseq_V8_Rev_94	CAAGCAGAAGACGGCATACGAGAT ATGCGAGACTTC GTGACTGGAGTTCAGACGTGTGCTCTTCCGATCT GACGGGCGGTGTGTRC	ATGCGAGACTTC
Miseq_V8_Rev_95	CAAGCAGAAGACGGCATACGAGAT CCGTCAAGATGT GTGACTGGAGTTCAGACGTGTGCTCTTCCGATCT TGACGGGCGGTGTGTRC	CCGTCAAGATGT
Miseq_V8_Rev_96	CAAGCAGAAGACGGCATACGAGAT TAGTAGCACCTG GTGACTGGAGTTCAGACGTGTGCTCTTCCGATCT CTGACGGGCGGTGTGTRC	TAGTAGCACCTG

V6-V8 region

Miseq reverse staggered refined primer list	
**JGI primer_ID**	**Sequence**
MiSeq_V6_fwd_spacer 0	AATGATACGGCGACCACCGAGATCTACAC TCTTTCCCTACA CGACGCTCTTCCGATCT AAACTYAAAKGAATTGRCGG
MiSeq_V6_fwd_spacer 3	AATGATACGGCGACCACCGAGATCTACAC TCTTTCCCTACA CGACGCTCTTCCGATCT GCTAAACTYAAAKGAATTGRCGG
MiSeq_V6_fwd_spacer 5	AATGATACGGCGACCACCGAGATCTACAC TCTTTCCCTACA CGACGCTCTTCCGATCT TGCGCAAACTYAAAKGAATTGRCGG
MiSeq_V6_fwd_spacer 6	AATGATACGGCGACCACCGAGATCTACAC TCTTTCCCTACA CGACGCTCTTCCGATCT CTGTGGAAACTYAAAKGAATTGRCGG
MiSeq_V6_fwd_random N	AATGATACGGCGACCACCGAGATCTACAC TCTTTCCCTACA CGACGCTCTTCCGATCT NNNNNAAACTYAAAKGAATTGRCGG

Miseq reverse staggered refined primer list		
**JGI primer_ID**	**Sequence**	**Barcode**
Miseq_V8_Rev_1	CAAGCAGAAGACGGCATACGAGAT TTACCGACGAGT GTGACTGGAGTTCAGACGTGTGCTCTTCCGATCT ACGGGCGGTGTGTRC	TTACCGACGAGT
Miseq_V8_Rev_2	CAAGCAGAAGACGGCATACGAGAT ATTGGACACGCT GTGACTGGAGTTCAGACGTGTGCTCTTCCGATCT GACGGGCGGTGTGTRC	ATTGGACACGCT
Miseq_V8_Rev_3	CAAGCAGAAGACGGCATACGAGAT TCGCATGGATAC GTGACTGGAGTTCAGACGTGTGCTCTTCCGATCT TGACGGGCGGTGTGTRC	TCGCATGGATAC
Miseq_V8_Rev_4	CAAGCAGAAGACGGCATACGAGAT AGCGAACCTGTT GTGACTGGAGTTCAGACGTGTGCTCTTCCGATCT CTGACGGGCGGTGTGTRC	AGCGAACCTGTT
Miseq_V8_Rev_5	CAAGCAGAAGACGGCATACGAGAT AGCTTCGACAGT GTGACTGGAGTTCAGACGTGTGCTCTTCCGATCT ACGGGCGGTGTGTRC	AGCTTCGACAGT
Miseq_V8_Rev_6	CAAGCAGAAGACGGCATACGAGAT GTCAGCCGTTAA GTGACTGGAGTTCAGACGTGTGCTCTTCCGATCT GACGGGCGGTGTGTRC	GTCAGCCGTTAA
Miseq_V8_Rev_7	CAAGCAGAAGACGGCATACGAGAT TCCAGATAGCGT GTGACTGGAGTTCAGACGTGTGCTCTTCCGATCT TGACGGGCGGTGTGTRC	TCCAGATAGCGT
Miseq_V8_Rev_8	CAAGCAGAAGACGGCATACGAGAT GAGAGTCCACTT GTGACTGGAGTTCAGACGTGTGCTCTTCCGATCT CTGACGGGCGGTGTGTRC	GAGAGTCCACTT
Miseq_V8_Rev_9	CAAGCAGAAGACGGCATACGAGAT GCTCACAATGTG GTGACTGGAGTTCAGACGTGTGCTCTTCCGATCT ACGGGCGGTGTGTRC	GCTCACAATGTG
Miseq_V8_Rev_10	CAAGCAGAAGACGGCATACGAGAT TTGACGACATCG GTGACTGGAGTTCAGACGTGTGCTCTTCCGATCT GACGGGCGGTGTGTRC	TTGACGACATCG
Miseq_V8_Rev_11	CAAGCAGAAGACGGCATACGAGAT CTTAGAACGTGC GTGACTGGAGTTCAGACGTGTGCTCTTCCGATCT TGACGGGCGGTGTGTRC	CTTAGAACGTGC
Miseq_V8_Rev_12	CAAGCAGAAGACGGCATACGAGAT CGGTTCACATAG GTGACTGGAGTTCAGACGTGTGCTCTTCCGATCT CTGACGGGCGGTGTGTRC	CGGTTCACATAG
Miseq_V8_Rev_13	CAAGCAGAAGACGGCATACGAGAT CGATAGGCCTTA GTGACTGGAGTTCAGACGTGTGCTCTTCCGATCT ACGGGCGGTGTGTRC	CGATAGGCCTTA
Miseq_V8_Rev_14	CAAGCAGAAGACGGCATACGAGAT GCTATATCCAGG GTGACTGGAGTTCAGACGTGTGCTCTTCCGATCT GACGGGCGGTGTGTRC	GCTATATCCAGG
Miseq_V8_Rev_15	CAAGCAGAAGACGGCATACGAGAT GTCTTCAGCAAG GTGACTGGAGTTCAGACGTGTGCTCTTCCGATCT TGACGGGCGGTGTGTRC	GTCTTCAGCAAG
Miseq_V8_Rev_16	CAAGCAGAAGACGGCATACGAGAT TAGACACCGTGT GTGACTGGAGTTCAGACGTGTGCTCTTCCGATCT CTGACGGGCGGTGTGTRC	TAGACACCGTGT
Miseq_V8_Rev_17	CAAGCAGAAGACGGCATACGAGAT TCAGCTGACTAG GTGACTGGAGTTCAGACGTGTGCTCTTCCGATCT ACGGGCGGTGTGTRC	TCAGCTGACTAG
Miseq_V8_Rev_18	CAAGCAGAAGACGGCATACGAGAT TAAGTCGGCCTA GTGACTGGAGTTCAGACGTGTGCTCTTCCGATCT GACGGGCGGTGTGTRC	TAAGTCGGCCTA
Miseq_V8_Rev_19	CAAGCAGAAGACGGCATACGAGAT GCTCCTTAGAAG GTGACTGGAGTTCAGACGTGTGCTCTTCCGATCT TGACGGGCGGTGTGTRC	GCTCCTTAGAAG
Miseq_V8_Rev_20	CAAGCAGAAGACGGCATACGAGAT GCTCCTTAGAAG GTGACTGGAGTTCAGACGTGTGCTCTTCCGATCT CTGACGGGCGGTGTGTRC	ATGGCCTGACTA
Miseq_V8_Rev_21	CAAGCAGAAGACGGCATACGAGAT TTGCAAGTACCG GTGACTGGAGTTCAGACGTGTGCTCTTCCGATCT ACGGGCGGTGTGTRC	TTGCAAGTACCG
Miseq_V8_Rev_22	CAAGCAGAAGACGGCATACGAGAT CCTAGTAAGCTG GTGACTGGAGTTCAGACGTGTGCTCTTCCGATCT GACGGGCGGTGTGTRC	CCTAGTAAGCTG
Miseq_V8_Rev_23	CAAGCAGAAGACGGCATACGAGAT CTAGGATCACTG GTGACTGGAGTTCAGACGTGTGCTCTTCCGATCT TGACGGGCGGTGTGTRC	CTAGGATCACTG

Miseq_V8_Rev_24	CAAGCAGAAGACGGCATACGAGAT TATGAACGTCCG GTGACTGGAGTTCAGACGTGTGCTCTTCCGATCT CTGACGGGCGGTGTGTRC	TATGAACGTCCG
Miseq_V8_Rev_25	CAAGCAGAAGACGGCATACGAGAT CTTGTGCGACAA GTGACTGGAGTTCAGACGTGTGCTCTTCCGATCT ACGGGCGGTGTGTRC	CTTGTGCGACAA
Miseq_V8_Rev_26	CAAGCAGAAGACGGCATACGAGAT CACGATGGTCAT GTGACTGGAGTTCAGACGTGTGCTCTTCCGATCT GACGGGCGGTGTGTRC	CACGATGGTCAT
Miseq_V8_Rev_27	CAAGCAGAAGACGGCATACGAGAT ACGTGCCTTAGA GTGACTGGAGTTCAGACGTGTGCTCTTCCGATCT TGACGGGCGGTGTGTRC	ACGTGCCTTAGA
Miseq_V8_Rev_28	CAAGCAGAAGACGGCATACGAGAT TGAACTAGCGTC GTGACTGGAGTTCAGACGTGTGCTCTTCCGATCT CTGACGGGCGGTGTGTRC	TGAACTAGCGTC
Miseq_V8_Rev_29	CAAGCAGAAGACGGCATACGAGAT TATTCAGCGGAC GTGACTGGAGTTCAGACGTGTGCTCTTCCGATCT ACGGGCGGTGTGTRC	TATTCAGCGGAC
Miseq_V8_Rev_30	CAAGCAGAAGACGGCATACGAGAT TAATCGGTGCCA GTGACTGGAGTTCAGACGTGTGCTCTTCCGATCT GACGGGCGGTGTGTRC	TAATCGGTGCCA
Miseq_V8_Rev_31	CAAGCAGAAGACGGCATACGAGAT GCGTCCATGAAT GTGACTGGAGTTCAGACGTGTGCTCTTCCGATCT TGACGGGCGGTGTGTRC	GCGTCCATGAAT
Miseq_V8_Rev_32	CAAGCAGAAGACGGCATACGAGAT CGTAAGATGCCT GTGACTGGAGTTCAGACGTGTGCTCTTCCGATCT CTGACGGGCGGTGTGTRC	CGTAAGATGCCT
Miseq_V8_Rev_33	CAAGCAGAAGACGGCATACGAGAT CTGTTACAGCGA GTGACTGGAGTTCAGACGTGTGCTCTTCCGATCT ACGGGCGGTGTGTRC	CTGTTACAGCGA
Miseq_V8_Rev_34	CAAGCAGAAGACGGCATACGAGAT ACGATCATCTGG GTGACTGGAGTTCAGACGTGTGCTCTTCCGATCT GACGGGCGGTGTGTRC	ACGATCATCTGG
Miseq_V8_Rev_35	CAAGCAGAAGACGGCATACGAGAT GTAACGGCTCTA GTGACTGGAGTTCAGACGTGTGCTCTTCCGATCT TGACGGGCGGTGTGTRC	GTAACGGCTCTA
Miseq_V8_Rev_36	CAAGCAGAAGACGGCATACGAGAT CCATGCTTAGAG GTGACTGGAGTTCAGACGTGTGCTCTTCCGATCT CTGACGGGCGGTGTGTRC	CCATGCTTAGAG
Miseq_V8_Rev_37	CAAGCAGAAGACGGCATACGAGAT GTACGCACAGTT GTGACTGGAGTTCAGACGTGTGCTCTTCCGATCT ACGGGCGGTGTGTRC	GTACGCACAGTT
Miseq_V8_Rev_38	CAAGCAGAAGACGGCATACGAGAT TTAGAGCCATGC GTGACTGGAGTTCAGACGTGTGCTCTTCCGATCT GACGGGCGGTGTGTRC	TTAGAGCCATGC
Miseq_V8_Rev_39	CAAGCAGAAGACGGCATACGAGAT ATAAGGTCGCCT GTGACTGGAGTTCAGACGTGTGCTCTTCCGATCT TGACGGGCGGTGTGTRC	ATAAGGTCGCCT
Miseq_V8_Rev_40	CAAGCAGAAGACGGCATACGAGAT AGTGGCACTATC GTGACTGGAGTTCAGACGTGTGCTCTTCCGATCT CTGACGGGCGGTGTGTRC	AGTGGCACTATC
Miseq_V8_Rev_41	CAAGCAGAAGACGGCATACGAGAT CCAGAAGTGTTC GTGACTGGAGTTCAGACGTGTGCTCTTCCGATCT ACGGGCGGTGTGTRC	CCAGAAGTGTTC
Miseq_V8_Rev_42	CAAGCAGAAGACGGCATACGAGAT CTACTAGCGGTA GTGACTGGAGTTCAGACGTGTGCTCTTCCGATCT GACGGGCGGTGTGTRC	CTACTAGCGGTA
Miseq_V8_Rev_43	CAAGCAGAAGACGGCATACGAGAT TAGCGTTCCAGA GTGACTGGAGTTCAGACGTGTGCTCTTCCGATCT TGACGGGCGGTGTGTRC	TAGCGTTCCAGA
Miseq_V8_Rev_44	CAAGCAGAAGACGGCATACGAGAT GTGAGTCATACC GTGACTGGAGTTCAGACGTGTGCTCTTCCGATCT CTGACGGGCGGTGTGTRC	GTGAGTCATACC
Miseq_V8_Rev_45	CAAGCAGAAGACGGCATACGAGAT TGGTCCTACAAG GTGACTGGAGTTCAGACGTGTGCTCTTCCGATCT ACGGGCGGTGTGTRC	TGGTCCTACAAG
Miseq_V8_Rev_46	CAAGCAGAAGACGGCATACGAGAT TACGCGTACAGT GTGACTGGAGTTCAGACGTGTGCTCTTCCGATCT GACGGGCGGTGTGTRC	TACGCGTACAGT
Miseq_V8_Rev_47	CAAGCAGAAGACGGCATACGAGAT GAGCCATCTGTA GTGACTGGAGTTCAGACGTGTGCTCTTCCGATCT TGACGGGCGGTGTGTRC	GAGCCATCTGTA
Miseq_V8_Rev_48	CAAGCAGAAGACGGCATACGAGAT CGTCCGTATGAA GTGACTGGAGTTCAGACGTGTGCTCTTCCGATCT CTGACGGGCGGTGTGTRC	CGTCCGTATGAA
Miseq_V8_Rev_49	CAAGCAGAAGACGGCATACGAGAT GATACGTTCGCA GTGACTGGAGTTCAGACGTGTGCTCTTCCGATCT ACGGGCGGTGTGTRC	GATACGTTCGCA
Miseq_V8_Rev_50	CAAGCAGAAGACGGCATACGAGAT CAGCTGGTTCAA GTGACTGGAGTTCAGACGTGTGCTCTTCCGATCT GACGGGCGGTGTGTRC	CAGCTGGTTCAA
Miseq_V8_Rev_51	CAAGCAGAAGACGGCATACGAGAT TTAAGCGCCTGA GTGACTGGAGTTCAGACGTGTGCTCTTCCGATCT TGACGGGCGGTGTGTRC	TTAAGCGCCTGA
Miseq_V8_Rev_52	CAAGCAGAAGACGGCATACGAGAT CCTGCGAAGTAT GTGACTGGAGTTCAGACGTGTGCTCTTCCGATCT CTGACGGGCGGTGTGTRC	CCTGCGAAGTAT
Miseq_V8_Rev_53	CAAGCAGAAGACGGCATACGAGAT TTGTAGCCGACA GTGACTGGAGTTCAGACGTGTGCTCTTCCGATCT ACGGGCGGTGTGTRC	TTGTAGCCGACA
Miseq_V8_Rev_54	CAAGCAGAAGACGGCATACGAGAT TCTGTAGAGCCA GTGACTGGAGTTCAGACGTGTGCTCTTCCGATCT GACGGGCGGTGTGTRC	TCTGTAGAGCCA
Miseq_V8_Rev_55	CAAGCAGAAGACGGCATACGAGAT CTATTAAGCGGC GTGACTGGAGTTCAGACGTGTGCTCTTCCGATCT TGACGGGCGGTGTGTRC	CTATTAAGCGGC
Miseq_V8_Rev_56	CAAGCAGAAGACGGCATACGAGAT CTCTGAGGTAAC GTGACTGGAGTTCAGACGTGTGCTCTTCCGATCT CTGACGGGCGGTGTGTRC	CTCTGAGGTAAC
Miseq_V8_Rev_57	CAAGCAGAAGACGGCATACGAGAT CAGGATTCGTAC GTGACTGGAGTTCAGACGTGTGCTCTTCCGATCT ACGGGCGGTGTGTRC	CAGGATTCGTAC
Miseq_V8_Rev_58	CAAGCAGAAGACGGCATACGAGAT TCACTGCTAGGA GTGACTGGAGTTCAGACGTGTGCTCTTCCGATCT GACGGGCGGTGTGTRC	TCACTGCTAGGA
Miseq_V8_Rev_59	CAAGCAGAAGACGGCATACGAGAT ACATGTCACGTG GTGACTGGAGTTCAGACGTGTGCTCTTCCGATCT TGACGGGCGGTGTGTRC	ACATGTCACGTG

Miseq_V8_Rev_60	CAAGCAGAAGACGGCATACGAGAT ATTCTGCCGAAG GTGACTGGAGTTCAGACGTGTGCTCTTCCGATCT CTGACGGGCGGTGTGTRC	ATTCTGCCGAAG
Miseq_V8_Rev_61	CAAGCAGAAGACGGCATACGAGAT TACACGCTGATG GTGACTGGAGTTCAGACGTGTGCTCTTCCGATCT ACGGGCGGTGTGTRC	TACACGCTGATG
Miseq_V8_Rev_62	CAAGCAGAAGACGGCATACGAGAT TGCATACACTGG GTGACTGGAGTTCAGACGTGTGCTCTTCCGATCT GACGGGCGGTGTGTRC	TGCATACACTGG
Miseq_V8_Rev_63	CAAGCAGAAGACGGCATACGAGAT ACGCAATGTCTG GTGACTGGAGTTCAGACGTGTGCTCTTCCGATCT TGACGGGCGGTGTGTRC	ACGCAATGTCTG
Miseq_V8_Rev_64	CAAGCAGAAGACGGCATACGAGAT GCTCGAAGATTC GTGACTGGAGTTCAGACGTGTGCTCTTCCGATCT CTGACGGGCGGTGTGTRC	GCTCGAAGATTC
Miseq_V8_Rev_65	CAAGCAGAAGACGGCATACGAGAT AGACGTTGCTAC GTGACTGGAGTTCAGACGTGTGCTCTTCCGATCT ACGGGCGGTGTGTRC	AGACGTTGCTAC
Miseq_V8_Rev_66	CAAGCAGAAGACGGCATACGAGAT TAGAGCTGCCAT GTGACTGGAGTTCAGACGTGTGCTCTTCCGATCT GACGGGCGGTGTGTRC	TAGAGCTGCCAT
Miseq_V8_Rev_67	CAAGCAGAAGACGGCATACGAGAT GGTAACCTCTGA GTGACTGGAGTTCAGACGTGTGCTCTTCCGATCT TGACGGGCGGTGTGTRC	GGTAACCTCTGA
Miseq_V8_Rev_68	CAAGCAGAAGACGGCATACGAGAT GACTTCATGCGA GTGACTGGAGTTCAGACGTGTGCTCTTCCGATCT CTGACGGGCGGTGTGTRC	GACTTCATGCGA
Miseq_V8_Rev_69	CAAGCAGAAGACGGCATACGAGAT CTGCATACTGAG GTGACTGGAGTTCAGACGTGTGCTCTTCCGATCT ACGGGCGGTGTGTRC	CTGCATACTGAG
Miseq_V8_Rev_70	CAAGCAGAAGACGGCATACGAGAT TAAGGCATCGCT GTGACTGGAGTTCAGACGTGTGCTCTTCCGATCT GACGGGCGGTGTGTRC	TAAGGCATCGCT
Miseq_V8_Rev_71	CAAGCAGAAGACGGCATACGAGAT AGTATTCGCGCA GTGACTGGAGTTCAGACGTGTGCTCTTCCGATCT TGACGGGCGGTGTGTRC	AGTATTCGCGCA
Miseq_V8_Rev_72	CAAGCAGAAGACGGCATACGAGAT TTCGCAGATACG GTGACTGGAGTTCAGACGTGTGCTCTTCCGATCT CTGACGGGCGGTGTGTRC	TTCGCAGATACG
Miseq_V8_Rev_73	CAAGCAGAAGACGGCATACGAGAT GCACCTGTTGAA GTGACTGGAGTTCAGACGTGTGCTCTTCCGATCT ACGGGCGGTGTGTRC	GCACCTGTTGAA
Miseq_V8_Rev_74	CAAGCAGAAGACGGCATACGAGAT CTCATGGTAGCA GTGACTGGAGTTCAGACGTGTGCTCTTCCGATCT GACGGGCGGTGTGTRC	CTCATGGTAGCA
Miseq_V8_Rev_75	CAAGCAGAAGACGGCATACGAGAT ACTAGTTGGACC GTGACTGGAGTTCAGACGTGTGCTCTTCCGATCT TGACGGGCGGTGTGTRC	ACTAGTTGGACC
Miseq_V8_Rev_76	CAAGCAGAAGACGGCATACGAGAT GCGGACTATTCA GTGACTGGAGTTCAGACGTGTGCTCTTCCGATCT CTGACGGGCGGTGTGTRC	GCGGACTATTCA
Miseq_V8_Rev_77	CAAGCAGAAGACGGCATACGAGAT ATCGCTTAAGGC GTGACTGGAGTTCAGACGTGTGCTCTTCCGATCT ACGGGCGGTGTGTRC	ATCGCTTAAGGC
Miseq_V8_Rev_78	CAAGCAGAAGACGGCATACGAGAT TCAGGACGTATC GTGACTGGAGTTCAGACGTGTGCTCTTCCGATCT GACGGGCGGTGTGTRC	TCAGGACGTATC
Miseq_V8_Rev_79	CAAGCAGAAGACGGCATACGAGAT GCATTACTGGAC GTGACTGGAGTTCAGACGTGTGCTCTTCCGATCT TGACGGGCGGTGTGTRC	GCATTACTGGAC
Miseq_V8_Rev_80	CAAGCAGAAGACGGCATACGAGAT GCTATGGAACTC GTGACTGGAGTTCAGACGTGTGCTCTTCCGATCT CTGACGGGCGGTGTGTRC	GCTATGGAACTC
Miseq_V8_Rev_81	CAAGCAGAAGACGGCATACGAGAT GATTGTGCAACC GTGACTGGAGTTCAGACGTGTGCTCTTCCGATCT ACGGGCGGTGTGTRC	GATTGTGCAACC
Miseq_V8_Rev_82	CAAGCAGAAGACGGCATACGAGAT AGCCTCATGATG GTGACTGGAGTTCAGACGTGTGCTCTTCCGATCT GACGGGCGGTGTGTRC	AGCCTCATGATG
Miseq_V8_Rev_83	CAAGCAGAAGACGGCATACGAGAT AACTCCTGTGGA GTGACTGGAGTTCAGACGTGTGCTCTTCCGATCT TGACGGGCGGTGTGTRC	AACTCCTGTGGA
Miseq_V8_Rev_84	CAAGCAGAAGACGGCATACGAGAT TAGAAGGCTCCT GTGACTGGAGTTCAGACGTGTGCTCTTCCGATCT CTGACGGGCGGTGTGTRC	TAGAAGGCTCCT
Miseq_V8_Rev_85	CAAGCAGAAGACGGCATACGAGAT GACTAGTCAGCT GTGACTGGAGTTCAGACGTGTGCTCTTCCGATCT ACGGGCGGTGTGTRC	GACTAGTCAGCT
Miseq_V8_Rev_86	CAAGCAGAAGACGGCATACGAGAT GGATACTCGCAT GTGACTGGAGTTCAGACGTGTGCTCTTCCGATCT GACGGGCGGTGTGTRC	GGATACTCGCAT
Miseq_V8_Rev_87	CAAGCAGAAGACGGCATACGAGAT CCGACATTGTAG GTGACTGGAGTTCAGACGTGTGCTCTTCCGATCT TGACGGGCGGTGTGTRC	CCGACATTGTAG
Miseq_V8_Rev_88	CAAGCAGAAGACGGCATACGAGAT TCGTGACGCTAA GTGACTGGAGTTCAGACGTGTGCTCTTCCGATCT CTGACGGGCGGTGTGTRC	TCGTGACGCTAA
Miseq_V8_Rev_89	CAAGCAGAAGACGGCATACGAGAT GGCCTATAAGTC GTGACTGGAGTTCAGACGTGTGCTCTTCCGATCT ACGGGCGGTGTGTRC	GGCCTATAAGTC
Miseq_V8_Rev_90	CAAGCAGAAGACGGCATACGAGAT GTAGCACTCATG GTGACTGGAGTTCAGACGTGTGCTCTTCCGATCT GACGGGCGGTGTGTRC	GTAGCACTCATG
Miseq_V8_Rev_91	CAAGCAGAAGACGGCATACGAGAT CTAAGACGTCGT GTGACTGGAGTTCAGACGTGTGCTCTTCCGATCT TGACGGGCGGTGTGTRC	CTAAGACGTCGT
Miseq_V8_Rev_92	CAAGCAGAAGACGGCATACGAGAT CGTGCACAATTG GTGACTGGAGTTCAGACGTGTGCTCTTCCGATCT CTGACGGGCGGTGTGTRC	CGTGCACAATTG
Miseq_V8_Rev_93	CAAGCAGAAGACGGCATACGAGAT TGTAACGCCGAT GTGACTGGAGTTCAGACGTGTGCTCTTCCGATCT ACGGGCGGTGTGTRC	TGTAACGCCGAT
Miseq_V8_Rev_94	CAAGCAGAAGACGGCATACGAGAT ATGCGAGACTTC GTGACTGGAGTTCAGACGTGTGCTCTTCCGATCT GACGGGCGGTGTGTRC	ATGCGAGACTTC
Miseq_V8_Rev_95	CAAGCAGAAGACGGCATACGAGAT CCGTCAAGATGT GTGACTGGAGTTCAGACGTGTGCTCTTCCGATCT TGACGGGCGGTGTGTRC	CCGTCAAGATGT

Miseq_V8_Rev_96	CAAGCAGAAGACGGCATACGAGAT TAGTAGCACCTG GTGACTGGAGTTCAGACGTGTGCTCTTCCGATCT CTGACGGGCGGTGTGTRC	TAGTAGCACCTG

Sequencing primer list

V4 region

Read 1 sequencing primer:

TCTTTCCCTACA GTGCCAGCMGCCGCGGTAA

Read 2 sequencing primer:

GTGACTGGAGTTCAGACGTGTGCTCTTCCGATCT

Index sequence primer:

GATCGGAAGAGCACACGTCTGAACTCCAGTCAC

V7-V8 region

Read 1 sequencing primer:

TCTTTCCCTACA GCAACGAGCGCAACCC

Read 2 sequencing primer:

GTGACTGGAGTTCAGACGTGTGCTCTTCCGATCT

Index sequence primer:

GATCGGAAGAGCACACGTCTGAACTCCAGTCAC

V6-V8 region

Read 1 sequencing primer:

TCTTTCCCTACA CGACGCTCTTCCGATCT

Read 2 sequencing primer:

GTGACTGGAGTTCAGACGTGTGCTCTTCCGATCT

Index sequence primer:

GATCGGAAGAGCACACGTCTGAACTCCAGTCAC

Itags amplification protocol

1. Illumina Itag PCR Conditions:

PCR Mastermix is prepared as below

Reagent
H20 (a)
5 Prime Hot MM (b)
Forward (10 uM) (c)
Reverse (10 uM) (c)
Template(~10ng/ul)

(a) PCR grade water was used
(b) 5 Prime Hot Master Mix was purchased from Fisher(Part # FP2200410).
(C) Primer concentrations are for the working stock.

2.Thermocyler conditions

Temp	Time
94°C	3 min.
94°C	45 sec.
50°C	1 min.
72°C	1.5 min.
72°C	10 min.
4°C	Hold

3.Protocol

a. Set up reactions in triplicates and amplify
b. Pool the triplicate amplified reactions
c. Clean up with 1.2X volume of AMPureXP® magnetic beads

d. Quantify amplicons with Qubit HS assay
e. Perform qualitative analysis using a Bioanalyzer (optional)
f. Pool amplicons in equal amounts.
g. Dilute the Pooled amplicons to 10nM and submit for qPCR
h. Sequence on Miseq; target 500K/mm2 cluster density and spike with approximately 25% Phix control
